# Molecular and clinical features of a Japanese medulloblastoma cohort: Subgroup‐specific prognostic stratification using economical/accessible diagnostic methods

**DOI:** 10.1111/bpa.70092

**Published:** 2026-03-11

**Authors:** Kohichi Go, Asako Katsuma, Ema Yoshioka, Tomoko Shofuda, Kohei Fukuoka, Koichi Ichimura, Yuko Matsushita, Yohei Mineharu, Yasuhide Makino, Takeshi Kawauchi, Atsushi Sasaki, Junko Hirato, Takeshi Inoue, Yoshinori Kodama, Masayuki Mano, Daisuke Kanematsu, Noriyuki Kijima, Naoki Kagawa, Dai Keino, Akitake Mukasa, Tomonari Suzuki, Koji Yoshimoto, Daisuke Kuga, Keishi Horiguchi, Shigeru Yamaguchi, Masayuki Kanamori, Kai Yamasaki, Kenichi Ishibashi, Takuya Akai, Masayoshi Yamaoka, Ryuji Ishizaki, Atsufumi Kawamura, Shigeo Ohba, Joji Ishida, Ryo Ando, Junya Fukai, Tomoru Miwa, Masazumi Fujii, Ai Muroi, Kuniaki Saito, Atsuko Harada, Yasuhiko Hayashi, Masahiro Nonaka, Young‐Soo Park, Yusuke Kobayashi, Tadashi Higuchi, Yosuke Miyairi, Kazuhisa Yoshifuji, Noriyoshi Takebe, Soichi Oya, Kosuke Nakajo, Mitsutoshi Nakada, Yoshiteru Nakano, Mizuki Kambara, Koji Adachi, Kazuhiro Tanaka, Hideo Nakamura, Yukihiko Sonoda, Ryuta Saito, Takafumi Wataya, Kazuhiko Kurozumi, Michael D. Taylor, Yoshitaka Narita, Soichiro Shibui, Hajime Arai, Hiroaki Sakamoto, Isao Date, Motoo Nagane, Ryo Nishikawa, Yoshiki Arakawa, Yonehiro Kanemura

**Affiliations:** ^1^ Department of Neurosurgery Graduate School of Medicine and Faculty of Medicine, Kyoto University Kyoto Japan; ^2^ Department of Biomedical Research and Innovation Institute for Clinical Research, NHO Osaka National Hospital Osaka Japan; ^3^ Department of Hematology/Oncology Saitama Children's Medical Center Saitama Japan; ^4^ Department of Pathology Kyorin University Faculty of Medicine Tokyo Japan; ^5^ Department of Pathology Saitama Medical University Saitama Japan; ^6^ Department of Pathology Public Tomioka General Hospital Gunma Japan; ^7^ Department of Pathology Osaka City General Hospital Osaka Japan; ^8^ Department of Central Laboratory and Surgical Pathology NHO Osaka National Hospital Osaka Japan; ^9^ Department of Diagnostic Pathology and Cytology Osaka International Cancer Institute Osaka Japan; ^10^ Department of Neurosurgery The University of Osaka Graduate School of Medicine Osaka Japan; ^11^ Department of Neurosurgery Yukioka Hospital Osaka Japan; ^12^ Division of Hematology/Oncology Kanagawa Children's Medical Center Kanagawa Japan; ^13^ Department of Neurosurgery Graduate School of Medical Sciences, Kumamoto University Kumamoto Japan; ^14^ Department of Pediatric Neuro‐Oncology/Neurosurgery Saitama Medical University International Medical Center Saitama Japan; ^15^ Department of Neurosurgery Graduate School of Medical Sciences, Kyushu University Fukuoka Japan; ^16^ Department of Neurosurgery Gunma University Graduate School of Medicine Gunma Japan; ^17^ Department of Neurosurgery Hokkaido University Graduate School of Medicine Hokkaido Japan; ^18^ Department of Neurosurgery Tohoku University Graduate School of Medicine Miyagi Japan; ^19^ Department of Pediatric Hematology and Oncology Osaka City General Hospital Osaka Japan; ^20^ Department of Neurosurgery Osaka City General Hospital Osaka Japan; ^21^ Department of Neurosurgery Graduate School of Medicine and Pharmaceutical Sciences, University of Toyama Toyama Japan; ^22^ Department of Pediatrics The Jikei University School of Medicine Tokyo Japan; ^23^ Department of Neurosurgery Shizuoka Children's Hospital Shizuoka Japan; ^24^ Department of Neurosurgery Hyogo Prefectural Kobe Children's Hospital Hyogo Japan; ^25^ Department of Neurosurgery Fujita Health University School of Medicine Aichi Japan; ^26^ Department of Neurological Surgery Okayama University Graduate School of Medicine, Dentistry and Pharmaceutical Sciences Okayama Japan; ^27^ Department of Neurosurgery Chiba Children's Hospital Chiba Japan; ^28^ Department of Neurological Surgery Wakayama Medical University School of Medicine Wakayama Japan; ^29^ Department of Neurosurgery Keio University School of Medicine Tokyo Japan; ^30^ Department of Neurosurgery Fukushima Medical University Fukushima Japan; ^31^ Department of Neurosurgery Institute of Medicine, University of Tsukuba Ibaraki Japan; ^32^ Department of Neurosurgery Kyorin University School of Medicine Tokyo Japan; ^33^ Department of Pediatric Neurosurgery Takatsuki General Hospital Osaka Japan; ^34^ Department of Neurosurgery Kanazawa Medical University Ishikawa Japan; ^35^ Department of Neurosurgery Kansai Medical University Osaka Japan; ^36^ Department of Neurosurgery and Children's Medical Center Nara Medical University Nara Japan; ^37^ Department of Neurosurgery Showa Medical University School of Medicine Tokyo Japan; ^38^ Department of Neurological Surgery Nippon Medical School Tokyo Japan; ^39^ Department of Neurosurgery Nagano Children's Hospital Nagano Japan; ^40^ Division of Pediatric Neurosurgery Hokkaido Medical Center for Child Health and Rehabilitation Hokkaido Japan; ^41^ Department of Neurosurgery Medical Research Institute Kitano Hospital, PIIF Tazuke‐kofukai Osaka Japan; ^42^ Department of Neurosurgery Saitama Medical Center, Saitama Medical University Saitama Japan; ^43^ Department of Neurosurgery Osaka Metropolitan University Graduate School of Medicine Osaka Japan; ^44^ Department of Neurosurgery Graduate School of Medical Science, Kanazawa University Ishikawa Japan; ^45^ Department of Neurosurgery School of Medicine, University of Occupational and Environmental Health Fukuoka Japan; ^46^ Department of Neurosurgery Shimane University Faculty of Medicine Shimane Japan; ^47^ Department of Neurological Surgery Nippon Medical School, Musashi‐Kosugi Hospital Kanagawa Japan; ^48^ Department of Neurosurgery Kobe University Graduate School of Medicine Hyogo Japan; ^49^ Department of Neurosurgery Kurume University School of Medicine Fukuoka Japan; ^50^ Department of Neurosurgery Yamagata University Faculty of Medicine Yamagata Japan; ^51^ Department of Neurosurgery Nagoya University, Graduate School of Medicine Aichi Japan; ^52^ Department of Neurosurgery Tokyo Metropolitan Children's Medical Center Tokyo Japan; ^53^ Department of Neurosurgery Hamamatsu University School of Medicine Shizuoka Japan; ^54^ Texas Children's Cancer and Hematology Center Houston Texas USA; ^55^ Department of Pediatrics–Hematology/Oncology Baylor College of Medicine Houston Texas USA; ^56^ Department of Neurosurgery Baylor College of Medicine Houston Texas USA; ^57^ Department of Neurosurgery Texas Children's Hospital Houston Texas USA; ^58^ Dan L Duncan Comprehensive Cancer Center Baylor College of Medicine Houston Texas USA; ^59^ The Arthur and Sonia Labatt Brain Tumour Research Centre and the Developmental and Stem Cell Biology Program The Hospital for Sick Children Toronto Ontario Canada; ^60^ Department of Surgery University of Toronto Toronto Ontario Canada; ^61^ Department of Laboratory Medicine and Pathobiology University of Toronto Toronto Ontario Canada; ^62^ Department of Medical Biophysics University of Toronto Toronto Ontario Canada; ^63^ Department of Neurosurgery and Neuro‐oncology National Cancer Center Hospital Tokyo Japan; ^64^ Department of Neurosurgery Teikyo University Hospital Mizonokuchi Japan; ^65^ Department of Neurosurgery Juntendo University Tokyo Japan; ^66^ Department of Pediatric Neurosurgery Osaka City General Hospital Osaka Japan; ^67^ Department of Neurosurgery NHO Osaka National Hospital Osaka Japan

**Keywords:** medulloblastoma, methylation analysis, molecular diagnosis, multiplex ligation‐dependent probe amplification, risk stratification

## Abstract

Medulloblastoma (MB) is a biologically and clinically heterogeneous pediatric brain tumor. However, large‐scale molecular subgrouping studies have mainly been conducted in Western populations, and comprehensive data from Asia are limited. To address this gap, we analyzed 242 MB cases collected from 39 institutions through the Japan Pediatric Molecular Neuro‐Oncology Group, performing centralized molecular classification using NanoString‐based gene expression profiling, DNA methylation arrays, and multiplex ligation‐dependent probe amplification (MLPA)‐based copy number profiling, supplemented by targeted sequencing. The subgroup distribution was 16.1% WNT, 24.8% SHH, 17.4% Group 3, and 41.7% Group 4. *CTNNB1* mutations and monosomy 6 characterized all WNT cases, whereas *MYCN* amplification and *TP53* mutations were independent adverse markers in SHH MB. Group 3 showed the worst survival, with *MYC* amplification and metastasis as poor prognostic factors. In Group 4, large cell/anaplastic histology predicted poor outcomes, whereas chromosome 11 loss was correlated with a favorable prognosis. Whole chromosomal aberration‐defined favorable‐risk patterns consistently indicate improved outcomes in non‐WNT/non‐SHH MBs. We also developed a simplified MLPA‐based classifier targeting six loci on chromosomes 7, 8, and 11 (SEE‐6‐CNA), which enabled robust and clinically feasible prognostic stratification. Overall, our findings confirm that the molecular subgroup‐specific features of Japanese MBs are largely concordant with global observations and that SEE‐6‐CNA provides a cost‐effective tool to support individualized treatment planning, particularly in resource‐limited settings.

## Graphical abstract

This study characterizes the molecular subgroup–specific features of a nationwide Japanese cohort comprising 242 medulloblastomas (MBs). Furthermore, we introduce SEE‐6‐CNA, a simple, cost‐effective, and FFPE‐compatible molecular test that is applicable to routine pathology samples and serves as a surrogate for risk stratification in non‐WNT/non‐SHH MBs.

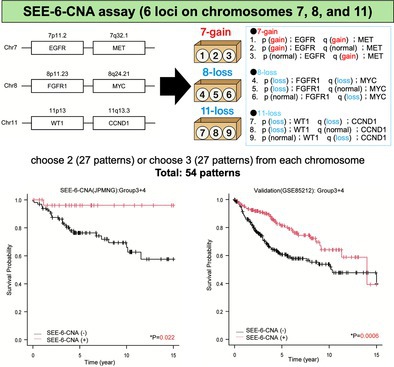

## 1 INTRODUCTION

Medulloblastoma (MB), traditionally classified as an embryonal tumor of the central nervous system (CNS), is the most common malignant pediatric brain tumor. According to recent epidemiological data, MB accounts for approximately 9–12% of CNS tumors in children, with age‐specific frequencies of 9.2% in the 0–4‐year group, 11.4% in the 5–9‐year group, and 5.8% in the 10–14‐year group, according to the CBTRUS 2017–2021 report. Similarly, the Kumamoto registry in Japan reported an incidence of 11% among pediatric CNS tumors. MB arises in the posterior fossa and exhibits a bimodal age distribution, with incidence peaks at 3–4 and 8–9 years of age, although adult cases have also been documented [1, 2, 3, 4]. MB arises in the posterior fossa and exhibits a bimodal age distribution, with incidence peaks at 3–4 and 8–9 years of age, although adult cases have been documented. Historically, MB has been designated as a World Health Organization (WHO) Grade IV malignancy because of its aggressive clinical behavior. However, recent advances in molecular profiling have significantly reshaped our understanding of its biological and clinical heterogeneity.

The integration of large‐scale genomic analyses and international collaborative efforts has led to the identification of distinct molecular subgroups with divergent clinical outcomes [5]. While extensive cohort studies with molecular subgrouping have been conducted in North America and Europe [5, 6], comparable datasets from other regions, including Asia, remain scarce, underscoring a critical geographic gap in our understanding of MB biology.

The current consensus stratifies MB into four biologically and clinically distinct subgroups: WNT‐activated (WNT), SHH‐activated (SHH), Group 3, and Group 4 [7, 8]. These subgroups differ in demographic distribution, histopathological characteristics, transcriptional profiles, and genomic alterations and are associated with markedly divergent prognoses [9]. This molecular classification framework has critical implications for clinical risk stratification and therapeutic decision making. Accordingly, the routine incorporation of molecular subgrouping into diagnostic workflows has been advocated. Several subgrouping methodologies have been developed, including NanoString‐based assays [10] and miRNA‐targeted real‐time PCR platforms [11, 12], both of which are compatible with formalin‐fixed, paraffin‐embedded specimens. DNA methylation profiling is widely regarded as the most robust method for molecular subgrouping and is recognized as the diagnostic gold standard in well‐resourced clinical and research settings [13].

Despite significant advances in molecular profiling, the global implementation of standardized MB subgrouping remains limited. The current WHO CNS tumor classification does not prescribe protocol‐level standards for MB subtyping, leading to considerable variability in diagnostic methodologies across institutions and geographic regions. Accurate molecular subtyping is essential for prognostic stratification and treatment planning in MB; however, routine implementation is often hindered by its cost and technical complexity. Although DNA methylation arrays offer highly informative and reproducible classifications, they are constrained by high costs, technical complexity, and extended turnaround times, limiting their feasibility for routine clinical use in several settings [14].

To address these challenges, we established the Japan Pediatric Molecular Neuro‐oncology Group (JPMNG), a collaborative initiative jointly led by the Japan Society of Neuro‐Oncology and the Japan Society of Pediatric Neurosurgery [15]. Using this effort, we assembled a nationwide retrospective cohort of patients with MB and conducted integrated analyses combining molecular subgrouping with pathological and clinical parameters. The primary objective was to assess the feasibility of a streamlined molecular classification within a real‐world, multi‐institutional framework and generate data that help rectify the current geographic under‐representation in global MB research. A novel feature of this study is the introduction of a simplified, cost‐effective copy number alteration (CNA)‐based classifier (SEE‐6‐CNA) derived from the multiplex ligation‐dependent probe amplification (MLPA) platform. This study represents the first nationwide effort in Japan to provide a practical alternative to array‐based profiling for non‐WNT/non‐SHH MB, enabling reliable subgroup classification in general hospital settings. Beyond its scale as the largest East Asian MB cohort analyzed to date, the significance of this study lies in translating subgroup‐specific biology into a widely applicable diagnostic strategy with direct clinical implications for risk stratification and patient management.

## 2 MATERIALS AND METHODS

### 2.1 Ethical considerations

This study was conducted in accordance with the principles of the Declaration of Helsinki. Ethical approval was obtained from the Institutional Review Board of Kyoto University Graduate School of Medicine (No. G0694), Osaka National Hospital (No. 13057), and all collaborating institutions. Written informed consent was obtained from all newly enrolled patients, whereas consent for past cases was acquired through an opt‐out process on the website. Individuals who declined to participate were excluded from the study.

### 2.2 Subjects, tumor samples, and central histopathological review

Patients with a histopathological diagnosis of MB between 1996 and 2018 at institutions or hospitals participating in the JPMNG were enrolled in this study. Tumor samples—including fresh, fresh‐frozen, and formalin‐fixed paraffin‐embedded specimens—along with clinical data from patients treated at affiliated institutions were collected.

All cases underwent central pathology review by three senior board‐certified neuropathologists (A.S., J.H., and K.I.) based on hematoxylin and eosin‐stained slides and additional immunohistochemical analyses (Data S1, Supporting Information). Consensus pathological diagnoses were made in accordance with the 2021 WHO classification of the CNS [16].

### 2.3 Molecular subgrouping

Molecular subgrouping was performed using the expression profiling of 22 MB subgroup‐specific genes with the NanoString nCounter system (NanoString Technologies Inc., Seattle, WA) [8] or genome‐wide DNA methylation profiling obtained via the Illumina Infinium HumanMethylationEPIC (EPIC array) or HumanMethylation450k (450 K array) BeadChip (Illumina, San Diego, CA) [17] (Data S1).

Molecular classification of DNA methylation profiles was conducted using the molecular classification algorithm from the German Cancer Center (DKFZ classifier, ver. 12.5). Methods for t‐distributed stochastic neighbor embedding (t‐SNE) of genome‐wide DNA methylation profiles have been previously described [13, 18].

### 2.4 DNA sequencing

The SNV status of *CTNNB1*, *TP53*, and the *TERT* promoter (*TERT*p) was determined using Sanger sequencing (Data S1). SNV statuses of *PTCH1*, *SUFU*, and *SMO* were also analyzed using next‐generation sequencing on an Ion Proton™ system (Thermo Fisher Scientific, Waltham, MA).

### 2.5 DNA copy number analysis

DNA copy number analysis was conducted using copy number variation (CNV) microarray platforms, including the Affymetrix CytoScan® 750K Array and CytoScan HD Array (Thermo Fisher Scientific), and the MLPA method (MRC Holland, Amsterdam, the Netherlands) (Data S1). The DNA copy number was calculated from the raw signal intensities of the genome‐wide DNA methylation array data obtained from the German Cancer Center website, as described above.

### 2.6 Validation analyses using public databases

Reference methylation data for MBs (GSE85218; 763 cases) [1] were obtained from the Gene Expression Omnibus database (http://www.ncbi.nlm.nih.gov/geo/) for comparative analyses. Molecular classification was performed using the German Cancer Center (DKFZ) classifier (version 12.5) (https://www.molecularneuropathology.org/mnp). In cases where the molecular subtype of Group 3/4 MBs showed discordance between the t‐SNE plot and classifier output, and the classifier's “calibrated score” was below 0.9, we prioritized t‐SNE analysis. This decision aligns with the classifier guidelines, which state that only predictions with calibrated scores above 0.9 are considered reliable.

### 2.7 Statistical analysis

Patient demographics, tumor characteristics, and treatment details were summarized using descriptive statistics, including medians with ranges, counts, and percentages for quantitative data. Subgroup comparisons were conducted using Pearson's chi‐squared test, Fisher's exact test, and Wilcoxon rank‐sum test. Overall survival (OS) was defined as the time until death from any cause. Survival curves were generated using the Kaplan–Meier method, and differences between patient groups were assessed using the log‐rank test and Cox proportional hazards modeling. All statistical analyses employed two‐sided tests, with *p*‐values less than 0.05 considered statistically significant. Data analysis was performed using R software (version 4.4.3) and JMP Pro 17 (SAS Institute, Inc., Cary, NC).

## 3 RESULTS

### 3.1 The JPMNG study cohort and its clinical and molecular characteristics

**FIGURE 1:**
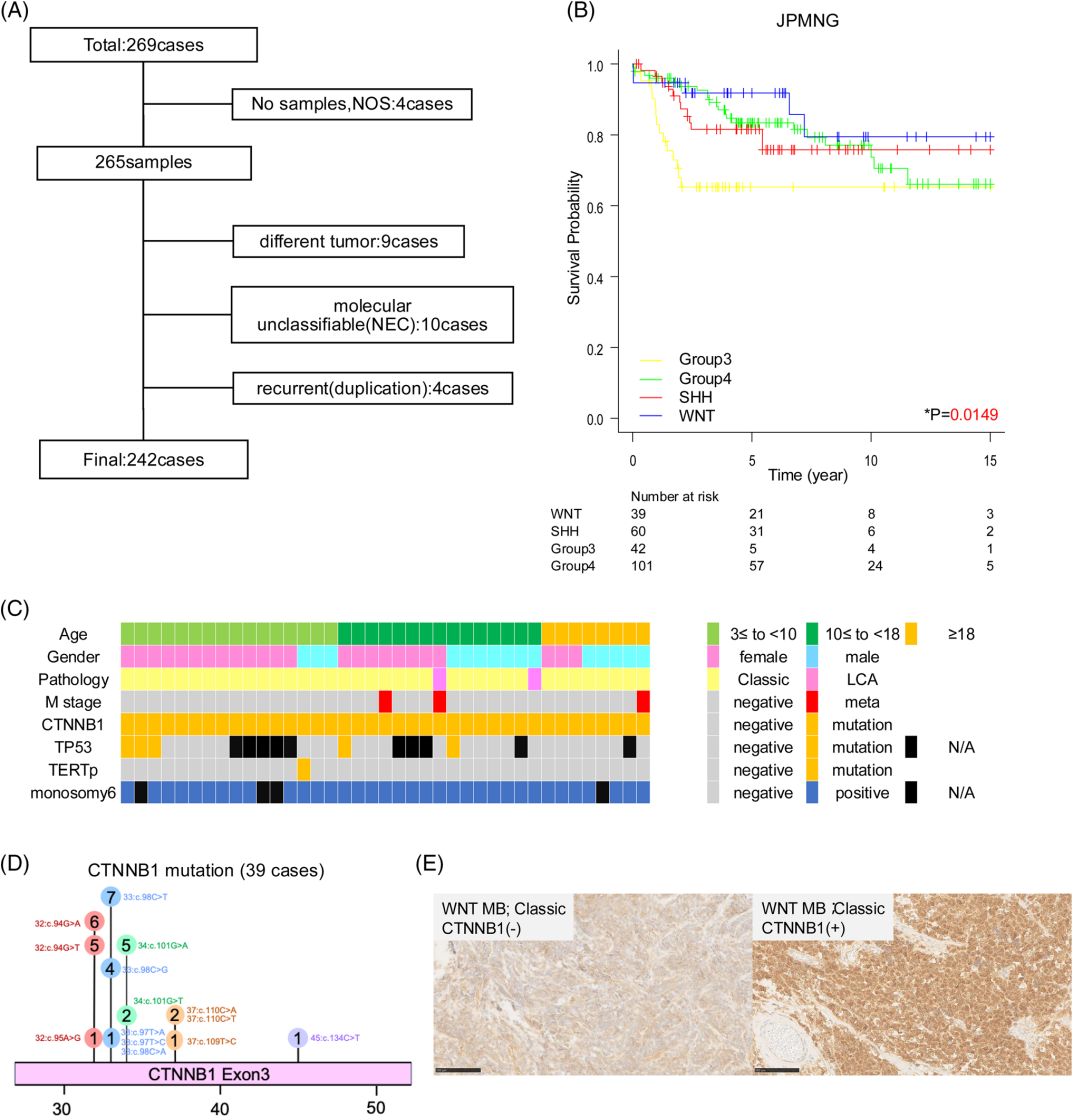
The JPMNG cohort and molecular and pathological characteristics of WNT subgroups. (A) Flowchart depicting case selection and exclusion criteria. (B) Kaplan–Meier survival analysis of the JPMNG cohort. Patients were classified into four molecular subgroups: WNT (blue), SHH (red), Group 3 (yellow), and Group 4 (green). Statistically significant survival differences were observed among the subgroups (log‐rank test, *p* = 0.0142). (C) Summary of the molecular and pathological characteristics of the WNT subgroup. (D) Distribution of *CTNNB1* mutations. Mutations were mapped across five hotspot regions within exon 3. The plot includes all 39 cases, with codon 33 variants (c.98C>A, c.97T>A, c.97T>C) occurring in one case each, and codon 37 variants (c.110C>T and c.110C>A) occurring in two cases each. (E) Representative β‐catenin immunohistochemistry in WNT subgroup cases. Nuclear β‐catenin accumulation was observed in 75% of the cases (right panel), whereas the remaining cases exhibited membranous staining without notable nuclear accumulation (left panel) (scale bar = 100 μm).

Initially, patients with MB (first surgery: 1996–2018) were registered at 39 institutes. Four cases were excluded due to insufficient specimens, and the remaining 265 underwent centralized histopathological and molecular evaluation. Subgrouping was performed using NanoString (265/265, 100%) and DNA methylation arrays (74/265, 27.9%) and Sanger sequencing (*CTNNB1*, *TP53*, *TERT*p) (Figure S1A). Following the exclusion of nine misdiagnosed cases, 10 Not Elsewhere Classified cases, and four relapse duplicates, survival analysis was conducted on 242 cases, comprising the JPMNG study cohort (Figures 1A and S1B). CNA profiling was conducted across all subgroups via methylation array and CNV microarray analysis, with additional subgroup‐specific testing using TaqMan (WNT) and MLPA (SHH, Group 3, and Group 4) (Figure S1A,B). The methylation array coverage in this cohort was 28.5% (69/242) (Figure S1C).

The JPMNG study cohort included 16.1% WNT, 24.8% SHH, 17.4% Group 3, and 41.7% Group 4 cases (Table 1). Compared with cohorts from North America and Europe, the proportion of the WNT phenotype was slightly higher and significantly greater than that in the MAGIC cohort (16.1% vs. 6.8%; *p* < 0.001). The proportion of SHH MBs was modestly lower than that in the MAGIC cohort (24.8% vs. 28.7%), although this difference was not statistically significant (*p* = 0.25). In contrast, the subgroup distribution showed no substantial differences compared to other East Asian cohorts (Figure S2A). The JPMNG cohort included all age groups, with approximately 50% pediatric cases (3 to <10 years) and 10% adults (≥18 years) (Table 1 and Figure S2B). No WNT cases were observed in infants (<3 years), of which approximately 60% were SHH. The frequency of WNT increased with age. Group 3 was frequent in infants and children, with only one adult case, whereas Group 4 peaked at 56.6% in adolescents (10 to <18 years) (Table 1 and Figure S2C). The WNT subgroup was absent in infants, and the proportion of SHH was relatively high in this age group. In children, Groups 3 and 4 collectively accounted for more than half of the cases, whereas in adults, WNT and SHH comprised over 60% of the cases (Figure S2D–F).

**TABLE 1:** JPMNG: Patient characteristics of MB subgroups (*N* = 242).

	WNT (*N* = 39; 16.1%)	SHH (*N* = 60; 24.8%)	Group 3 (*N* = 42; 17.4%)	Group 4 (*N* = 101; 41.7%)	Total (*N* = 242)
Age (years)					
Mean age (years)	13.2 (±7.71)	7.6 (±7.65)	5.8 (±4.18)	10.9 (±9.31)	9.53
Median age (years)	10.65	4.67	4.92	9.19	8.05
Range	5–40	0–31	1–24	1–69	
Infant (<3)	0 (0%)	23 (38.3%)	10 (23.8%)	4 (3.9%)	37 (15.3%)
Pediatric (≤3 to <10)	16 (41.0%)	22 (36.7%)	27 (64.3%)	54 (53.5%)	119 (49.2%)
Adolescent (≤10 to <18)	16 (41.0%)	7 (11.7%)	4 (9.5%)	35 (34.7%)	62 (25.6%)
Adult (≥18)	7 (18.0%)	8 (13.3%)	1 (2.4%)	8 (7.9%)	24 (9.9%)
Gender					
Male	15 (38.5%)	32 (53.3%)	25 (59.5%)	60 (59.4%)	132 (54.5%)
Female	24 (61.5%)	28 (46.7%)	17 (40.5%)	41 (40.6%)	110 (45.5%)
Histology					
Classic	37 (94.9%)	23 (38.3%)	35 (83.3%)	95 (94.0%)	190 (78.5%)
D/N	0 (0%)	21 (35.0%)	0 (0%)	0 (0%)	21 (8.7%)
MBEN	0 (0%)	10 (16.7%)	0 (0%)	0 (0%)	10 (4.1%)
LCA	2 (5.1%)	3 (5.0%)	6 (14.3%)	3 (3.0%)	14 (5.8%)
NOS, unknown	0 (0%)	3 (5.0%)	1 (2.4%)	3 (3.0%)	7 (2.9%)
Metastasis at diagnosis					
Negative	36 (92.3%)	44 (73.3%)	20 (47.6%)	69 (68.3%)	169 (69.8%)
Positive	3 (7.7%)	16 (26.7%)	22 (52.4%)	32 (31.7%)	73 (30.2%)

Abbreviations: D/N, desmoplastic/nodular; LCA, large cell/anaplastic; MBEN, medulloblastoma with extensive nodularity; WNT, Wingless signaling‐activated; SHH, Sonic‐hedgehog signaling‐activated.

The prognosis across the four molecular subgroups showed significant variations (*p* = 0.0149, log‐rank test). Five‐year overall survival (OS) rates were as follows: WNT (91.9%), SHH (81.7%), Group 3 (65.4%), and Group 4 (83.6%). The 10‐year overall survival rates were as follows: WNT (79.7%), SHH (75.8%), Group 3 (65.4%), and Group 4 (77.0%), indicating that WNT had the best outcome and Group 3 had the poorest outcome. Late‐onset progression was suspected in Group 4 (Figure 1B). Kaplan–Meier analysis by age showed significantly worse survival in infants (Group 3) (*p* = 0.00261, log‐rank test; Figure S3A). In this infant cohort, craniospinal irradiation (CSI) was administered to 3 of 23 SHH patients (13.0%) and 5 of 10 Group 3 patients (50.0%). Recurrence occurred in 6/10 (60.0%) Group 3 infants and metastatic disease at diagnosis (M+ at diagnosis) was present in 8/10 (80.0%), compared with 4/23 (17.4%) and 4/23 (17.4%), respectively, in SHH infants. These differences in disease status at diagnosis and during follow‐up likely contributed to the markedly poorer survival observed in Group 3 infants. However, older age groups showed no significant differences (Figure S3B–D). In the pediatric Group 4 cohort (3 to <10 years), 15 of 54 patients experienced tumor relapse, and M+ at diagnosis was observed in 12 of 54 patients, and five of these patients exhibited both M+ disease at diagnosis and subsequent tumor relapse. These high‐risk characteristics likely contributed to the late events observed beyond 5 years (Figure S3B). Age alone did not affect the prognosis of the cohort (Figure S3E). However, histopathological classification revealed significant variations in survival (*p* < 0.001; Figure S3F). Although metastasis did not reach statistical significance (*p* > 0.05), a trend toward poorer survival was noted in metastatic cases (Figure S3G).

### 3.2 WNT subgroup

A total of 39 WNT MB cases were diagnosed using NanoString, with DNA methylation validation in eight cases (Figure S1B). Female predominance was significant (61.5%), and most patients were aged 6–17 years (79.5%). Metastasis was rare (7.7%), as shown in Table 1. Histopathologically, 94.9% of the patients had classic MB (CMB), and two had large cell/anaplastic (LCA) MB. All cases harbored exon 3 *CTNNB1* mutations (Figure 1C), consistent with the known hotspots (Figure 1D), although nuclear β‐catenin positivity via immunostaining remained at 75% (9/12; Figure 1E). Monosomy 6 was confirmed in 35 tested cases via methylation array and/or TaqMan assay (Figure S4A,B). One *TERT*p mutation was found in all 39 cases (2.5%), and five *TP53* mutations were found among 29 samples (17.2%) (Figure 1C).

The five‐year OS rate was 93.7% in pediatric cases and 85.6% in adults, with no significant difference (*p* = 0.683; Figure S5A). *TP53* pathogenic variants, metastasis, and LCA were not statistically associated with poor prognosis, possibly due to the limited sample size (Figure S5B–D).

Overall, JPMNG WNT MB shares clinical and molecular features with prior reports, occurs mainly in females over 3 years of age, and is associated with favorable outcomes.

### 3.3 SHH subgroup

A total of 60 SHH MB cases were diagnosed using NanoString, with 17 confirmed by DNA methylation arrays (28.3%) (Table 1 and Figure S1B). CNAs were analyzed using MLPA (11 cases) and CNV microarray (22 cases) (Figure S1B). Most cases occurred in males under 10 years of age, especially under 3 (38.3%) and 3–10 (36.7%) years of age. The desmoplastic/nodular (35%) and MB with extensive nodularity (16.7%) subtypes were exclusive to SHH MB (Table 1).

**FIGURE 2:**
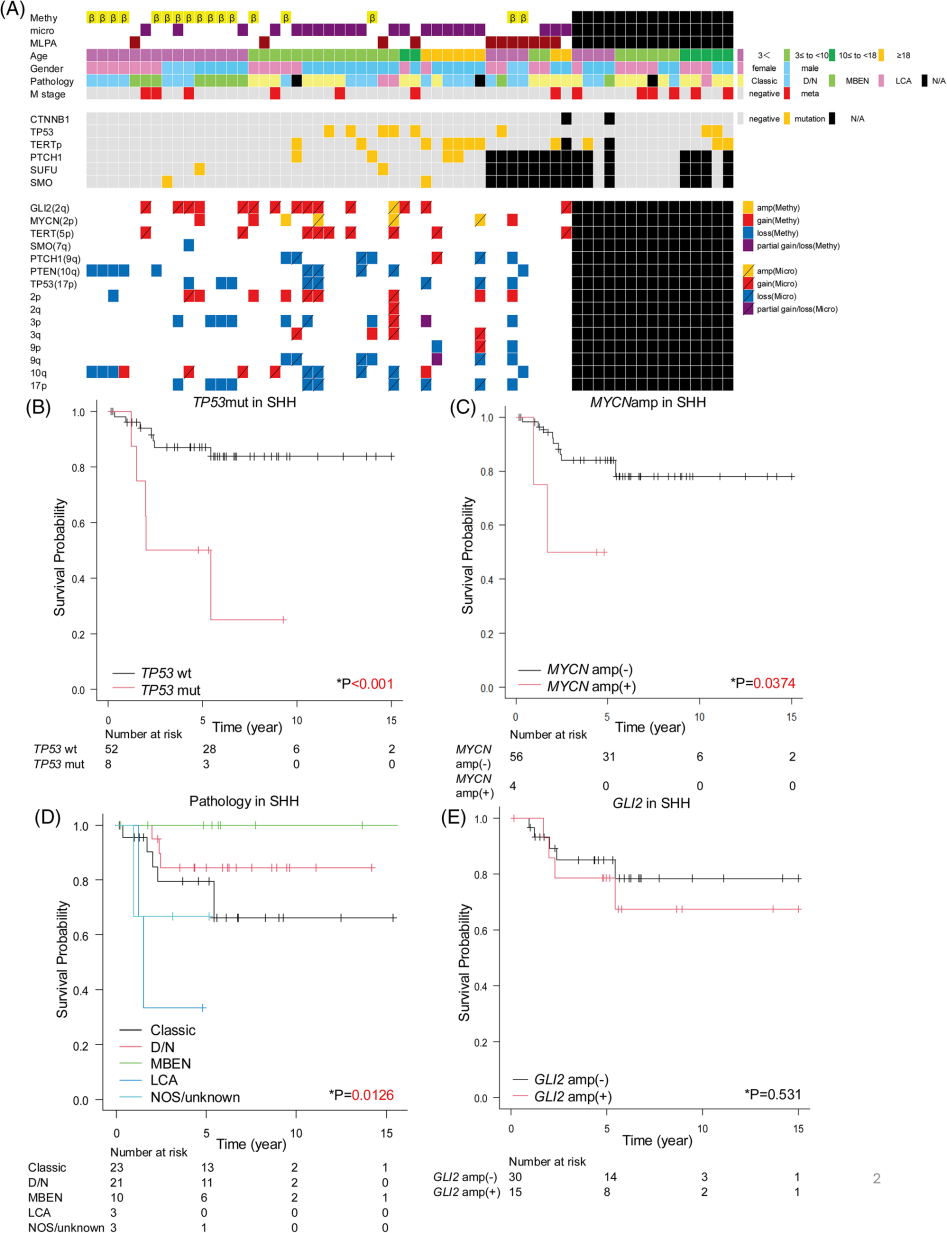
Integrated Molecular and Clinical Profiling of SHH MBs. (A) SHH MBs were classified using NanoString gene expression profiling, methylation array analysis (DKFZ classifier), and histopathological evaluation of the samples. Genetic analysis findings and clinical data were integrated to construct a comprehensive molecular and clinical profile of the patients. All SHH cases were classified as subtype “B” based on the methylation array results. Fifteen cases were excluded from the analysis due to insufficient sample quantity and are depicted in grayscale. Methy, methylation array; micro, microarray. (B) Kaplan–Meier overall survival curves stratified by *TP53* mutation status in SHH MBs. (C) Overall survival curves based on the *MYCN* copy number status in SHH MBs. (D) Overall survival curves according to the histopathological classification of SHH MBs. (E) Overall survival curves stratified by GLI2 amplification status in SHH MBs.

No *CTNNB1* mutations were found, but 11 *TERT*p mutations were identified (13.8%; Table S1), mostly in adults. *TP53* mutations were detected in 12.1% (8/58, Table S1) and CNAs in 17.8% (8/45; Figure 2A) of patients. These *TP53‐*mutated cases were distributed across age groups as follows: 0 in infants, 4 in pediatric patients, 3 in adolescents, and 1 in adults. Next‐generation sequencing revealed nine *PTCH1*, two *SUFU*, and two *SMO* mutations. *MYCN* amplification occurred in 8.9% (4/45) of patients, with one co‐occurrence with *TP53* mutation (2.2%; Figure 2A and Table S1). The frequencies were consistent with those reported by Northcott et al. [18], although *PTCH1*, *TERT*p, *SUFU*, and *SMO* mutations were less common (Table S2).

The prognosis was worse for cases with *TP53* mutations or *MYCN* amplification, and the histopathological subtype also affected prognosis (Figure 2B–D). *GLI2* amplification and factors such as age, *TERT*p, *PTCH1*, *SMO* mutations, and *TP53* loss were not significantly associated with outcomes (Figures 2E and S6A–E). *SUFU* mutations showed a trend toward poor prognosis (*p* = 0.0649), likely because of the small sample size (Figure S6F).

In summary, the JPMNG SHH MB cohort shares key clinical and prognostic features with previous reports, although minor interethnic genetic heterogeneity may be present.

### 3.4 Group 3 subgroup

A total of 42 cases were diagnosed with Group 3 MB using the NanoString platform, with DNA methylation array validation available for 17 cases (40.5%). Among these, six cases were assigned to subtype II, three to subtype III, seven to subtype IV, and one to subtype VII (Table 1 and Figure S1B). CNAs were assessed in 37 cases (88.1%) using MLPA, and microarray analysis was performed in six cases (Figure S1B). Importantly, no cases in our Group 3 cohort demonstrated loss of chromosome 8q based on MLPA or CNV microarray analyses; therefore, survival analysis of this alteration could not be performed. These cases predominantly occurred in male patients, with the most frequent age at diagnosis being 3–10 years (64.3%), followed by <3 years (23.8%). Overall, 80% of the cases were diagnosed in children under the age of 10 years, with only one adult case (2.4%) identified (Table 1). Histopathologically, the majority of tumors were classified as CMB (35/42; 83.3%), while six cases (14.3%) were designated as LCA subtype, which had the highest percentage among the remaining subtypes (Table 1). Metastatic disease was observed in 22 cases (22/42; 52.4%), representing the only subgroup with a notably high incidence of metastatic lesions (Table 1).

In the subset of Group 3 cases with available MLPA‐based copy‐number data (*n* = 37), age‐stratified analyses showed that M^+^ at diagnosis was observed in 5/7 (<3 years), 13/25 (3–10 years), 1/4 (10–18 years), and 0/1 (>18 years). *MYC* amplification demonstrated a similar pattern, being detected in 1/7 (<3 years), 5/25 (3–10 years), 0/4 (10–18 years), and 0/1 (>18 years). In our cohort, all infants (<3 years) with M+ at diagnosis received CSI. Notably, among the five infants with M+ at diagnosis, only one case without *MYC* amplification survived. These findings should be interpreted cautiously, given the small sample size and treatment heterogeneity in this retrospective cohort (Table S3).

**FIGURE 3:**
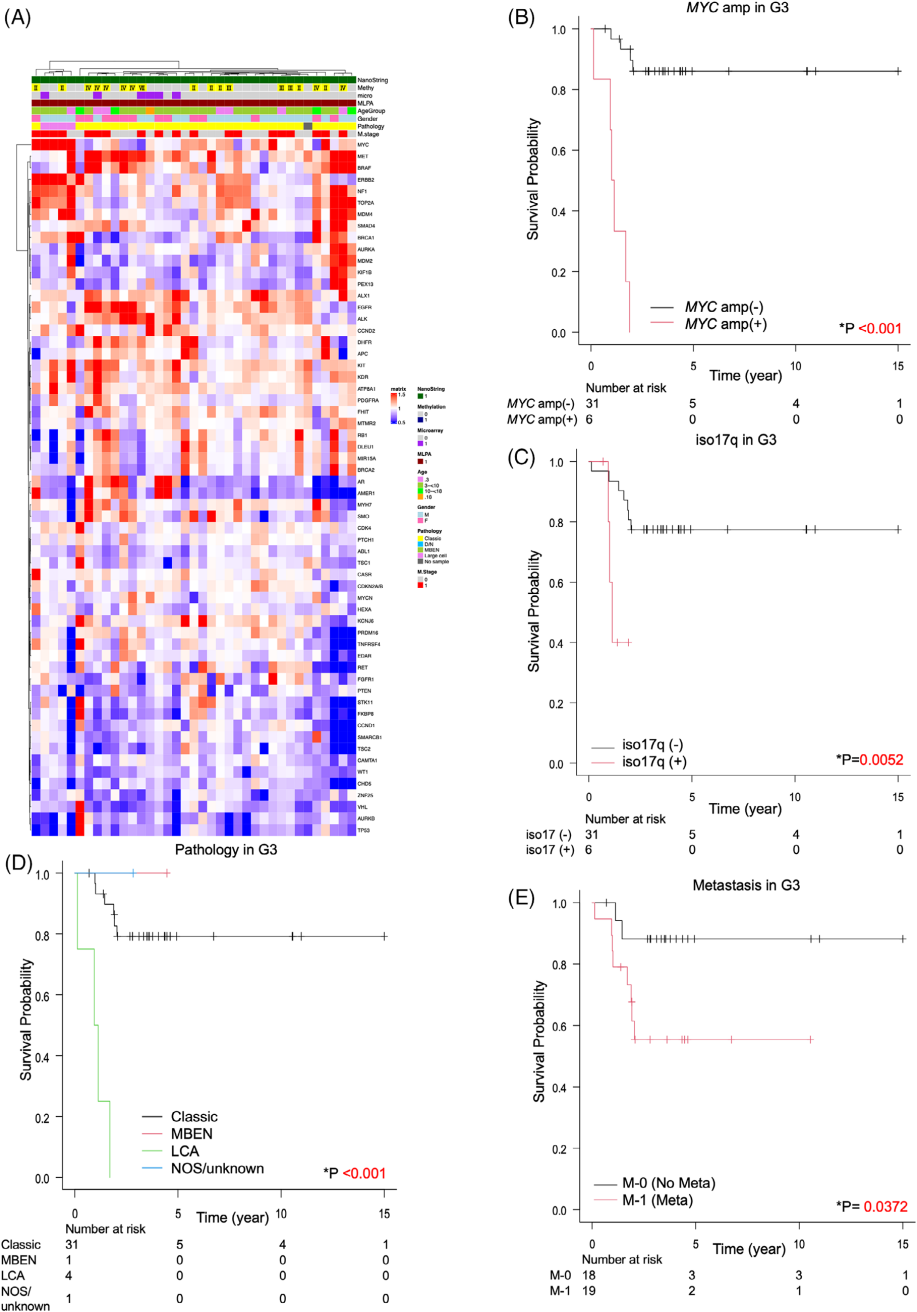
Integrated Molecular and Clinical Characterization of Group 3 MBs. (A) Group 3 MBs were classified using NanoString gene expression profiling, methylation array analysis (DKFZ classifier), and histopathological evaluation. Genetic analysis findings and clinical data were integrated to construct a comprehensive molecular and clinical profile. CNA profiles derived from MLPA are shown for Group 3 cases. Each row corresponds to a gene‐specific probe, and each column represents an individual tumor. Red indicates copy number gain, and blue indicates loss. Top panel annotations include the molecular classification method, MLPA availability, age group, gender, histological subtype, and metastatic stage. Cases classified by methylation profiling are highlighted in yellow, with the corresponding subtypes indicated in Roman numerals. Methy, methylation array; micro, microarray. (B) Kaplan–Meier overall survival curves stratified by *MYC* amplification status in Group 3 MB. (C) Overall survival curves according to isochromosome 17q (iso17q) status in Group 3 MB. (D) Survival analysis based on the histopathological classification of Group 3 MB. (E) Overall survival curves according to the metastatic status in Group 3 MB.

Cases with *MYC* amplification or isochromosome 17q (i17q: characterized by loss of 17p and/or gain of 17q) demonstrated significantly poorer prognoses than those without these alterations (*p* < 0.001, Figure 3B; *p* = 0.0052, Figure 3C). MLPA and microarray analyses confirmed that i17q was distinct from global chromosomal gains or losses, specifically exhibiting a segmental loss of 17p or gain at 17q (Figure S7A). Prognosis was significantly stratified by histopathological subtype, with LCA showing the poorest outcome (*p* < 0.001, Figure 3D). Additionally, metastatic status was strongly associated with an unfavorable prognosis (*p* = 0.0372, Figure 3E). In contrast, age at diagnosis was not significantly correlated with clinical outcomes (*p* = 0.934, Figure S7B).

Overall, our findings indicate that JPMNG Group 3 MB exhibits clinical characteristics consistent with previously reported cohorts and that key prognostic molecular markers are preserved across diverse ethnic populations.

### 3.5 Group 4 subgroup

**FIGURE 4:**
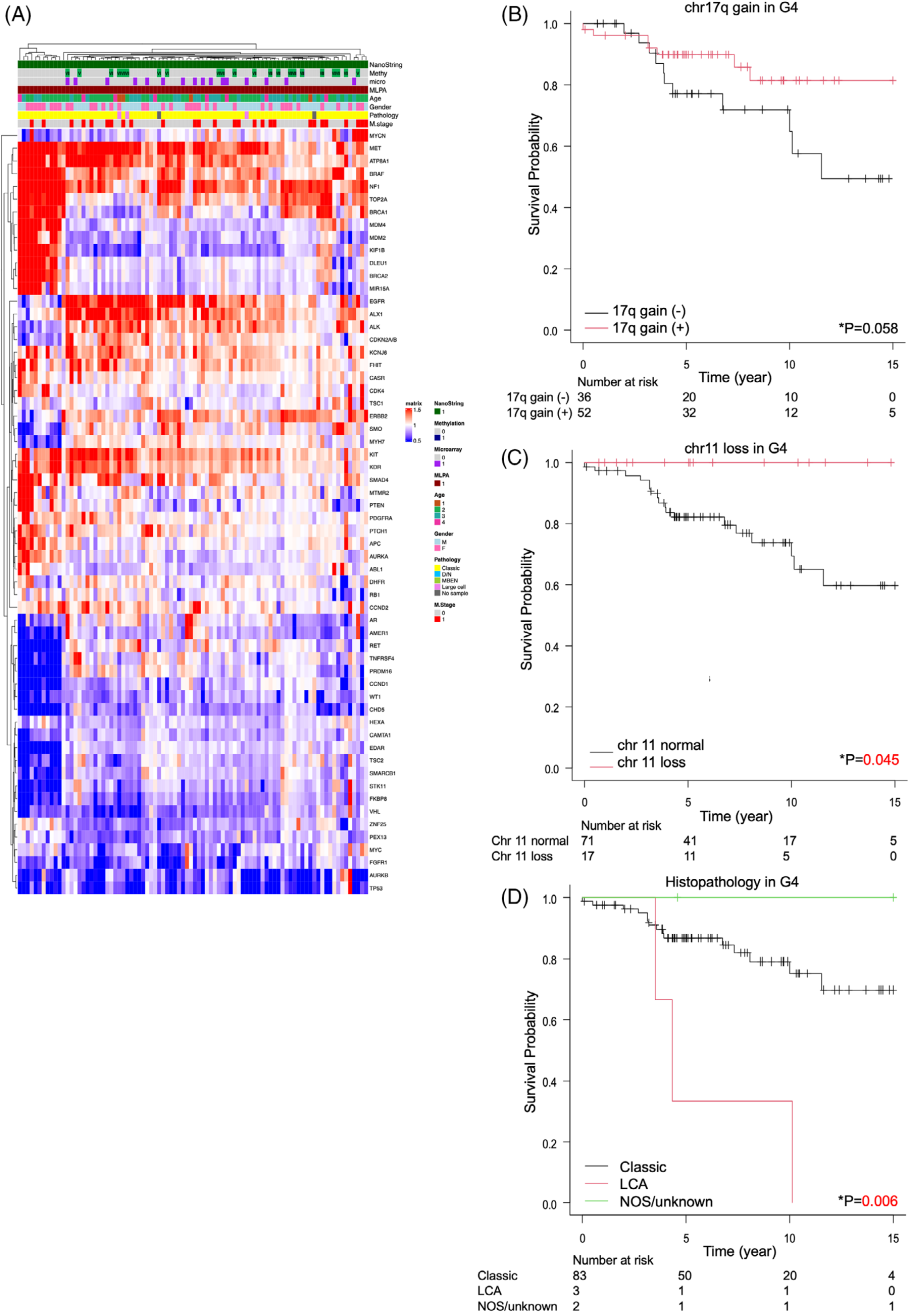
Integrated Molecular and Clinical Characterization of Group 4 MBs. (A) Group 4 MBs were classified using NanoString gene expression profiling, methylation array analysis (DKFZ classifier), and histopathological evaluation. Genetic analysis findings and clinical data were integrated to construct a comprehensive molecular and clinical profile. CNA profiles derived from MLPA analysis are shown for Group 4 cases. Each row corresponds to a gene‐specific probe, while each column represents an individual tumor. Red indicates copy number gain; blue indicates loss. Top panel annotations include molecular classification method, MLPA availability, age group, gender, histological subtype, and metastatic stage. Cases classified by methylation profiling are highlighted in yellow, with corresponding subtypes indicated in Roman numerals. Methy, methylation array; micro, microarray. (B) Kaplan–Meier overall survival curves stratified by chromosome 17q status in Group 4 MB. (C) Overall survival curves according to chromosome 11 status in Group 4 MB. (D) Survival analysis based on the histopathological classification of Group 4 MB.

A total of 101 cases were diagnosed as Group 4 MB using NanoString, with validation by DNA methylation array performed in 22 (21.8%) cases. Subtype classification identified two cases as subtype V, two as subtype VI, twelve as subtype VII, and six as subtype VIII (Figures 4A and S1B and Table 1). CNA analysis was conducted using MLPA in 91 (90.1%) and CNV microarray in 16 (15.8%) cases (Figure S1B). The majority of cases occurred in males, with the most frequent age at diagnosis between 3 and 10 years (53.5%), followed by 10–18 years of age (34.7%). Overall, 80% of the cases were diagnosed between the ages of 3 and 18 years (Table 1). Histopathologically, most cases were classified as classic CMB (96/101; 95.0%), while three (3.0%) were identified as LCA, the most common non‐classic subtype (Table 1). Metastasis was identified in 32 patients (31.7%), representing the group with the highest number of lesions (Table 1).

Cases with 17q gain had substantially better prognoses (Figure 4B), and those with chromosome (chr) 11 loss had significantly better prognoses than those without this alteration (*p* = 0.0052, Figure 4C). The 17q gain sample, confirmed by MLPA, involved a gain of 17q alone and was clearly distinguished from i17q and total chr 17 gains by CNV microarray results (Figure S7A). The prognosis was significantly stratified according to histopathological classification. Although limited by sample size, patients with LCA histology demonstrated notably poorer outcomes (Figure 4D). In contrast, neither metastatic status nor *MYCN* amplification was significantly associated with prognosis (Figure S7C,D). Whole‐chromosome 17 gain was not observed in the JPMNG cohort; all chr17 abnormalities consisted of 17q gains accompanied by 17p loss. Therefore, survival analysis based on whole‐chromosome 17 gain could not be performed in this study.

### 3.6 A novel prognostic stratification approach for non‐WNT/non‐SHH medulloblastoma

The non‐WNT/non‐SHH subgroup of MB is characterized by marked heterogeneity, encompassing both favorable and unfavorable prognoses. Numerous studies have sought to establish risk stratification in this subgroup of patients. Goschzik et al. [19] proposed a prognostic classification based on Whole Chromosomal Aberration (WCA) profiling using microarray technology. In our current analysis of the JPMNG cohort, we evaluated WCA patterns using DNA methylation and SNP microarray data and assessed their prognostic relevance. Cases within the non‐WNT/non‐SHH subgroup exhibiting WCA favorable‐risk (WCA‐FR) patterns consistently demonstrated improved prognosis (Figure S8A). In Group 3, two patients with WCA‐FR patterns experienced no deaths, although statistical significance was not achieved, likely because of the limited sample size. In contrast, Group 4 cases with WCA‐FR patterns showed significantly better overall survival (Figure S8B,C). These findings suggest that WCA‐FR patterns are prognostically informative in the JPMNG cohort.

**FIGURE 5:**
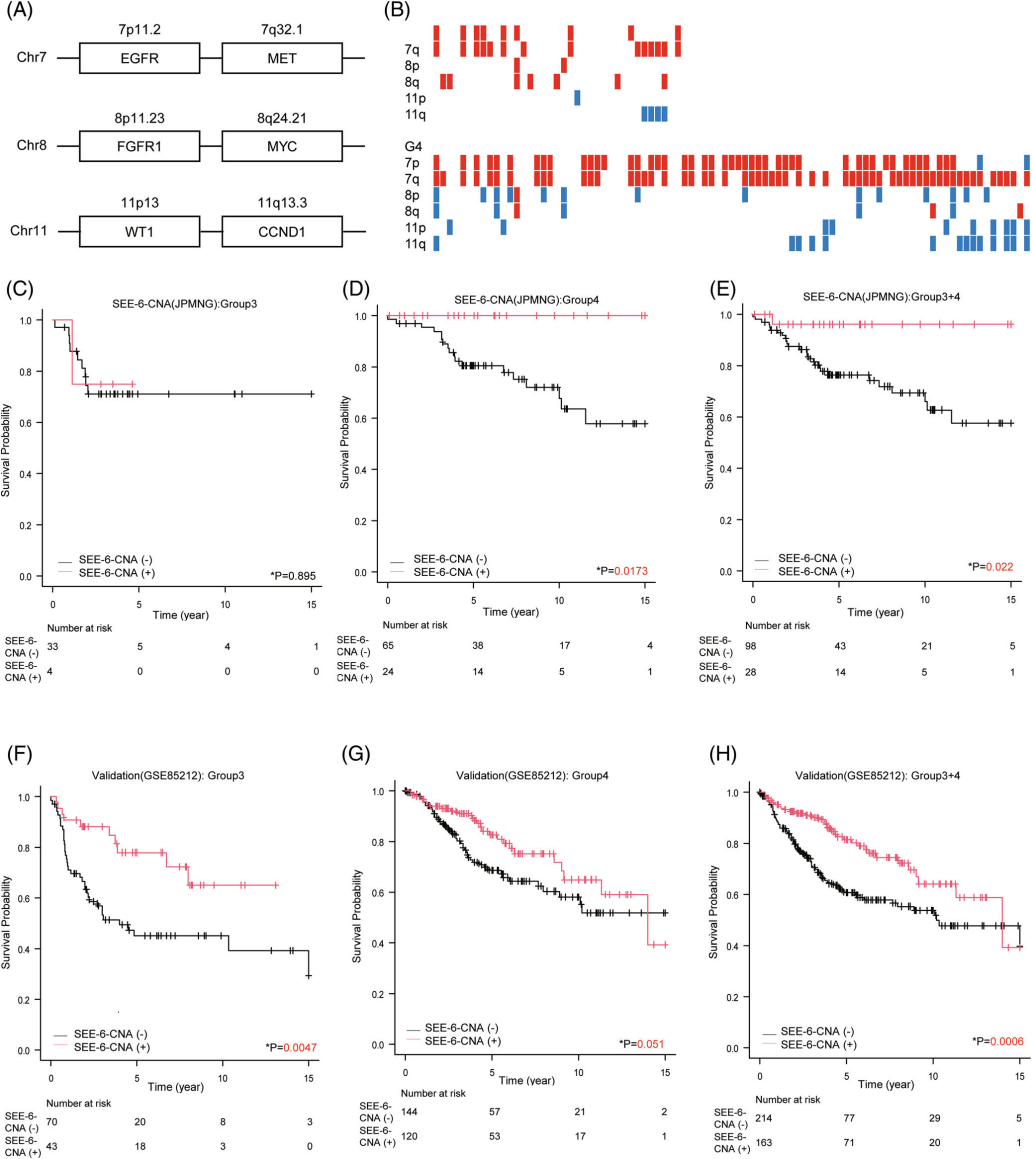
Definition and Distribution of Copy Number–based WCA‐like Patterns via MLPA Analysis. (A) Copy number alterations were assessed across the selected chromosomal loci using MLPA. Six gene‐specific probes—targeting 7p (*EGFR*), 7q (*MET*), 8p (*FGFR1*), 8q (*MYC*), 11p (*WT1*), and 11q (*CCND1*)—were analyzed to identify WCA‐FR‐like patterns, defined by the presence of at least one copy number gain on chromosome 7 and one loss on chromosomes 8 or 11. A total of 54 distinct non‐redundant alteration patterns were identified. (B) Heatmap displaying copy number alterations (CNAs) across individual tumor samples. Each column represents a tumor, and each row corresponds to one of the six target loci. Copy number gain (≥1.3) is shown in red, loss (≤0.5) in blue, and neutral copy number (0.5–1.3) is unshaded. (C) Kaplan–Meier overall survival curves for Group 3 MBs in the JPMNG Discovery cohort. (D) Overall survival curves for JPMNG Group 4 MBs in the JPMNG Discovery cohort. (E) Overall survival curves for combined Group 3 and Group 4 MBs in the JPMNG Discovery cohort. (F) Overall survival curves for Group 3 MBs in the GSE85212 validation cohort. (G) Overall survival curves for Group 4 MBs in the GSE85212 validation cohort. (H) Overall survival curves for combined Group 3 and Group 4 MBs in the GSE85212 validation cohort.

To establish an alternative surrogate method for identifying WCA‐FR patterns within the non‐WNT/non‐SHH MB population, we assessed the copy number status of six marker genes located on the short (p) and long (q) arms of three chromosomes using the MLPA platform. Based on this analysis, we propose a novel concept, SEE‐6‐CNA. SEE‐6‐CNA refers to CNAs involving six marker genes located on three chromosomes: 7 (S: seven), 8 (E: eight), and 11 (E: eleven). Specifically, the affected loci included *EGFR* (7p), *MET* (7q), *FGFR1* (8p), *MYC* (8q), *WT1* (11p), and *CCND1* (11q) (Figure 5A). Unlike conventional WCA criteria, which require concordant aberrations across chromosomal arms, we independently evaluated gains and losses for each arm; for example, alterations in 7p and 7q were assessed separately (Figure 5B).

Theoretically, 54 distinct CNA pattern combinations can be derived: 27 from two‐chromosome combinations and 27 from three‐chromosome combinations (Figure S8D). In practice, one such pattern was observed in Group 3 (four cases), whereas 12 unique patterns encompassing 24 cases were identified in Group 4 (Figure S8E). Although individual patterns lacked sufficient statistical power, the combined analysis of these 24 cases revealed a significantly prolonged OS compared with cases without such combinations (Figure 5C–E).

To validate our findings, we analyzed 763 cases of MB from the GSE85212 dataset [1]. After excluding cases with incomplete survival data, 377 cases (Group 3: *n* = 113; Group 4: *n* = 264) were included in the survival analysis (Figure S8F). Among the 246 Group 4 cases, 60 exhibited one of the nine WCA‐FR patterns defined by the SEE‐6‐CNA model, whereas the remaining three patterns were not represented in this dataset. Notably, these 60 Group 4 cases showed significantly improved survival, mirroring the trends observed in the JPMNG cohort (*p* = 0.024, Figure S8G). We also conducted separate analyses for Group 3 and a combined analysis of Groups 3 and 4, confirming significant survival differences across all comparisons (Figure 5F–H).

Radiotherapy data were available for most non‐WNT/non‐SHH patients. The distribution of treatment modalities did not differ substantially between SEE‐6‐CNA‐positive and ‐negative groups. In Group 3, CSI was delivered in 75.0% (3/4) of SEE‐6‐CNA‐positive cases and 87.9% (29/33) of SEE‐6‐CNA‐negative cases, with similar proportions of low‐, standard‐, and high‐dose CSI between the two groups. In Group 4, CSI was delivered in 95.8% (23/24) of SEE‐6‐CNA‐positive cases and 90.8% (59/65) of SEE‐6‐CNA‐negative cases, again without a clear shift toward reduced or high‐dose CSI in either group. These findings indicate that heterogeneity in radiotherapy intensity is unlikely to account for the survival differences associated with the SEE‐6‐CNA (Table S4).

These findings suggest that WCA‐FR patterns are valuable for prognostic evaluation in the non‐WNT/non‐SHH subgroup of MB. Moreover, assessment via SEE‐6‐CNA serves as an effective surrogate for WCA‐FR patterns and enables meaningful risk stratification within this heterogeneous group of patients.

## 4 DISCUSSION

MB is one of the most common pediatric brain tumors in Japan. According to population‐based registry data, the incidence of pediatric MB among children aged 0–14 years in Japan was 0.39 per 100,000 person‐years in 2016 [20]. This rate is comparable to estimates from the United States (approximately 0.39–0.48 per 100,000, depending on the dataset and time period) [21] but lower than those reported in Europe for the same age group (~6.8 per million) [22]. In this nationwide, multi‐institutional, retrospective study of MB cases registered at 39 institutes across Japan, we conducted comprehensive molecular diagnostics and survival analyses, revealing distinct clinical and prognostic features according to the molecular subgroups. All cases were classified using NanoString‐based gene expression profiling, with consistency validated by DNA methylation profiling of a subset.

Compared with Western cohorts, the JPMNG cohort showed a slightly higher proportion of WNT and a slightly lower proportion of SHH subgroups (Figure S2). However, the overall subgroup distribution was largely consistent with that of other East Asian cohorts [5, 23, 24, 25, 26]. Age‐specific patterns—such as the absence of WNT in infants and Group 4 predominance in adolescents—highlight the interplay between molecular biology and age in disease behavior. While histopathology stratified prognosis, age alone did not, underscoring the clinical utility of molecular classification.

Overall, our findings demonstrate that the molecular epidemiology of MB in Japan aligns broadly with global data, while also revealing distinct regional features. These results reinforce the importance of molecular classification‐based risk stratification and support the development of tailored treatment strategies in Japanese clinical settings.

WNT MB is typically driven by *CTNNB1* exon 3 mutations, with monosomy 6 frequently co‐occurring in these patients [5, 18, 27, 28, 29]. In our cohort, both alterations were observed in 100% of evaluable WNT cases, suggesting high diagnostic reliability. However, previous studies have reported *CTNNB1*‐wildtype and non‐WNT tumors with monosomy 6, indicating that these markers are not entirely subgroup‐specific [29, 30, 31]. Although IHC for nuclear β‐catenin demonstrates high specificity (99.23%), its limited sensitivity (76.2%) may result in the missed detection of a subset of WNT MBs [24, 32]. In this study, we observed discrepancies between NanoString‐based classification and DNA methylation profiling, highlighting the limitations of relying on a single diagnostic modality. To address this issue, we propose a stepwise, clinically practical, and cost‐effective diagnostic strategy for settings in which access to DNA methylation arrays is limited. From an economic standpoint, the MLPA‐based copy number assay used in this workflow is relatively low‐cost: based on current manufacturer list prices, the combined probemix and reagent kit correspond to roughly US$15–20 in reagents per sample, and in our institutional setting the total running cost remains on the order of US$30–40 per assay, with a hands‐on time of approximately 1 h. The approach begins with initial molecular classification using NanoString, followed by *CTNNB1* sequencing or β‐catenin IHC. WNT MB is diagnosed if either *CTNNB1* mutation or nuclear β‐catenin positivity is detected. If both are negative, monosomy 6 status is assessed; a positive result suggests a likely WNT subtype. In cases where all preceding tests are negative, DNA methylation profiling should be employed to guide the final diagnosis (Figure S9).

The lower 10‐year OS observed in our WNT cohort should be interpreted with caution, as follows: follow‐up completeness markedly decreased beyond 5 years, resulting in limited evaluable long‐term survivors. Furthermore, the two deaths that occurred after 5 years were attributable to suicide and a non‐tumor‐related medical condition rather than MB progression, which may have contributed to the modest decline in the 10‐year survival estimate. Standard treatment practices for WNT MB in Japan remained largely unchanged during the study period, although international clinical trends gradually moved toward reduced‐dose radiation during the 2010s. Although this de‐escalation was not widely adopted in Japan, minor institutional variations may have modestly influenced long‐term survival estimates.

SHH MB arises from the dysregulation of the SHH signaling pathway, which plays a pivotal role in cellular development [33]. Compared to the findings of Northcott et al. [18], our cohort exhibited similar frequencies of *TP53* mutations and *MYCN* amplifications. SHH MB is biologically heterogeneous and has been classified into four subtypes based on DNA methylation profiling [1]. In our cohort, methylation array analysis revealed an exclusive SHH‐β classification, likely reflecting the predominance of infant cases (82.4%). *TERT* promoter mutations, which are typically observed in adults, were consistent with previous reports [34]. These findings suggest that the core molecular features of SHH MB are preserved in the JPMNG cohort, underscoring the biological relevance of these alterations.

Genomic profiling has revealed mutually exclusive somatic mutations in *PTCH1* [35], *SUFU* [36], and *SMO* [37]. However, the prevalence of *PTCH1* mutations was notably lower (20% vs. 43%), whereas *PTEN* alterations were higher (22.2% vs. 7%), potentially reflecting ethnic or population‐specific differences. No significant prognostic impact was observed for mutations in *PTCH1*, *SUFU*, or *SMO* (Figure S6D–F). These deviations from widely recognized global patterns warrant validation in larger and more ethnically diverse cohorts.

Zhukova et al. [38] reported that *TP53* mutations are associated with poor prognosis in SHH MBs, but not in WNT MBs, with most affected patients dying within 2 years of their diagnosis. Consequently, SHH MBs harboring *TP53* mutations are classified as a distinct entity in the WHO classification and have been proposed to represent a “very high‐risk” subgroup [39]. Furthermore, *MYCN* amplification has been identified as a poor prognostic marker [6, 30, 40]. In the present study, SHH MBs with either *TP53* mutations or *MYCN* amplification exhibited significantly worse survival, consistent with previous reports [6, 30, 40, 41], suggesting that both alterations are critical risk factors for SHH MBs.

The incidence of *GLI2* amplification was higher in our cohort (15/45) than in previous reports, including large cytogenetic series of SHH MB [5]. This may reflect the larger proportion of infant and early childhood SHH cases in our cohort, differences in CNV detection platforms, and potential interethnic variation. GLI2 amplification was not associated with survival in our series, possibly because co‐occurring TP53 mutations and/or MYCN amplification, which are molecular features that define high‐risk SHH subtypes and have been linked to poor prognosis in previous studies, were relatively rare [38, 39].

*TP53* mutations and *MYCN* amplification occasionally co‐occur in SHH MBs and are established markers of extremely poor prognosis [42]. However, in the JPMNG cohort, the overlap between these alterations was rare, with only one case exhibiting both alterations. The remaining cases harbored either *TP53* mutations or *MYCN* amplification alone and consistently demonstrated poor clinical outcomes, suggesting that each alteration independently contributes to aggressive disease behavior. This observation aligns with the findings of Mitani et al. [43], who reported that *MYCN* amplification, even in the absence of *TP53* mutations, is associated with a poor prognosis, likely driven by underlying genomic instability. The potential association between *TP53* mutations and *MYCN* amplification warrants further investigation in future studies.

Groups 3 and 4 collectively accounted for approximately 65% of all MB cases and exhibited distinct clinical characteristics and outcomes. Established high‐risk factors include age <3 years at diagnosis, metastatic disease, *MYC* amplification, and LCA histology [19, 44]. Moreover, three independent studies have demonstrated that Groups 3 and 4 MBs can be further subdivided into multiple subtypes based on their DNA methylation profiles. Subsequent meta‐analyses reconciled these findings into eight clinically meaningful, second‐generation methylation subtypes (I–VIII) spanning both groups [45, 46].

In the present study, i17q and *MYC* amplifications were significantly associated with poor prognosis in Group 3 MBs, consistent with previous reports (Figure 3) [3]. Our cohort corroborated these associations, with three out of seven *MYC*‐amplified cases falling into subgroup II, in agreement with the findings of Sharma et al. [45]. Furthermore, metastatic disease was linked to poorer outcomes, and *MYC* amplification was observed more frequently among metastatic cases (6/22, 27%), although it did not represent the majority of cases. Notably, high‐risk cases were found in subgroups II, III, IV, and VII, whereas no *MYC* amplification was observed in the prognostically favorable subgroups V, VI, and VIII (Figure 3). LCA histology was also significantly associated with poor prognosis (Figures 3 and S7), underscoring its universal relevance for risk stratification in this subgroup.

In Group 4, LCA histology was significantly associated with poor prognosis, and chr 11 loss was significantly associated with good prognosis. Although the limited number of cases may have contributed to the lack of statistical significance, cases with chr 17q gain also demonstrated a trend toward a favorable prognosis (Figure 4). These findings suggest that these molecular and histological features serve as robust prognostic indicators for Group 4 MBs. Conversely, neither metastatic disease nor *MYCN* amplification was significantly associated with prognosis, underscoring the ongoing controversy regarding the clinical relevance of these factors.

Previous large‐scale clinical trials, including SJMB03 and ACNS0331, reported that neither isochromosome 17q nor LCA histology had a significant impact on the outcome in Group 4 MB [47, 48]. In contrast, the size of our Group 4 cohort, particularly the number of LCA cases (*n* = 5), was comparatively small, and our findings regarding these markers should therefore be regarded as exploratory. The discrepancies between these studies and our findings may reflect differences in cohort composition, ethnic background, and relative frequencies of specific molecular alterations. In the JPMNG cohort, LCA histology and certain chromosomal abnormalities (such as chromosome 11 loss and 17q gain) were less common but showed clearer prognostic separation, suggesting that their biological impacts may vary across populations. These observations highlight the heterogeneity of Group 4 MB and underscore the importance of validating prognostic markers across diverse ethnic datasets.

Risk stratification strategies for non‐WNT/non‐SHH MBs, particularly Group 4 MBs, continue to generate strong interest because of the persistent risk of late‐onset recurrence or progression of these tumors. The eight‐subtype classification has greatly contributed to risk evaluation in non‐WNT/non‐SHH MBs; however, access to DNA methylation array‐based diagnostics remains limited in most general hospital settings [49, 50]. As an alternative approach, Goddard et al. [51] retrospectively analyzed the molecular profiles of patients enrolled in clinical trials conducted across European countries and identified the WCA‐FR signature—defined by the presence of two or more chromosomal aberrations among chr 7 gain, chr 8 loss, and chr 11 loss—as being strongly associated with a favorable prognosis. Furthermore, integrating the WCA‐FR signature with the eight‐subtype classification enabled a more refined stratification of Group 4 MB patients, highlighting the potential of these novel diagnostic approaches for risk assessment in this subgroup of patients.

Diagnosis of the WCA‐FR signature also requires microarray‐based diagnostic methods, similar to DNA methylation arrays, and these biomarkers are not readily accessible in routine clinical settings. As a surrogate approach, we developed a method for more accessible and cost‐effective detection of CNA using the MLPA platform. The newly developed SEE‐6‐CNA was significantly associated with a favorable prognosis in Group 4 MBs within the JPMNG cohort, and its robustness was further supported by validation using the publicly available large‐scale GSE85212 dataset (Figure 5) [1].

The MLPA system is a widely used method for determining CNA and is generally compatible with standard capillary DNA sequencing [52, 53]. It is technically straightforward, economically efficient, and suitable for implementation in general hospitals. Accordingly, the SEE‐6‐CNA assay based on MLPA is highly likely to be adopted in clinical settings and may substantially contribute to broader risk assessment efforts for Group 4 MBs.

This study had some limitations. First, as is commonly encountered in pediatric tumor samples, the amount of tissue obtained through surgical resection or biopsy is often limited. Consequently, insufficient DNA and RNA yields limit the feasibility of some molecular analyses. In particular, CNA analysis could not be conducted in some cases, and methylation array‐based validation was not feasible for all samples. Furthermore, *APC* mutation testing was not performed and, therefore, not included in our diagnostic workflow. Second, risk stratification was not adjusted according to the treatment modalities. Future studies should aim to incorporate multidisciplinary treatment classifications and conduct prospective clinical trials to validate the prognostic implications of these findings. In addition, the applicability of the SEE‐6‐CNA classifier to Group 3 MB remains uncertain because the number of SEE‐6‐CNA‐positive Group 3 patients in our cohort was extremely small (*n* = 4). This limited sample size reduced the statistical robustness of subgroup‐specific survival estimates. Although a similar favorable trend was observed in the GSE85212 dataset, larger international cohorts are required to determine whether SEE‐6‐CNA has prognostic relevance in Group 3 disease.

Although the magnitude of the survival difference was smaller in the GSE85212 dataset than in the JPMNG cohort, this is likely due to methodological differences in how chromosome copy number alterations were inferred, as CNV status in GSE85212 was estimated from methylation array data using analytical thresholds not fully specified in the original study. Such platform‐dependent variability may attenuate survival separation; however, the consistent trend across cohorts supports the robustness and generalizability of the SEE‐6‐CNA markers.

In addition, although we examined the relevance of the cytogenetic risk model proposed by Shih et al., fully reproducing their stratification scheme was not feasible because their framework relied on a broader set of chromosomal alterations derived from high‐resolution array‐based CNV profiling. As our study focused on a limited set of six loci detectable by MLPA, the variables used did not completely overlap, and the applicability of the full Shih model to our dataset remained inherently constrained.

Finally, potential biases related to the retrospective nature of the study and inter‐institutional variability in sample handling may have influenced the data quality and completeness. Furthermore, although our follow‐up period was sufficient for preliminary survival analysis, long‐term monitoring is essential to validate subgroup‐specific prognostic markers, particularly for late‐stage events.

## 5 CONCLUSION

In this nationwide study conducted by the JPMNG, we analyzed the molecular genetic profiles of 242 MB cases and identified clinically relevant subgroup‐specific features, most of which were consistent with previously reported findings. To further refine risk stratification, we applied MLPA to detect CNAs following molecular subgrouping of patients. As a surrogate marker for WCA‐FR patterns, we developed a simplified and cost‐effective method, termed SEE‐6‐CNA, which was significantly associated with the prognosis of Group 4 MBs. These molecular diagnostic approaches are expected to improve accessibility to risk assessment in general hospitals and significantly contribute to the implementation of MB risk stratification in routine clinical practice.

## AUTHOR CONTRIBUTIONS

*Conceptualization*: Y. Kanemura, Y.A., S.S., H.A., H.S., I.D., M. Nagane, R.N. *Methodology*: K.G., A. Katsuma, E.Y., T. Shofuda, K. F., K. Ichimura, Y. Matsushita, D. Kanematsu, A.S., J.H., T.I., Y. Kodama, M.M., M.D.T., and Y. Kanemura. *Validation*: K.G., A. Katsuma, E.Y., K.F., Y.A., and Y. Kanemura. *Formal analysis*: K.G., A. Katsuma, E.Y., Y. Kanemura. *Investigation*: K.G., A. Katsuma, E.Y., T. Shofuda, K.F., K. Ichimura, Y. Matsushita, D. Kanematsu. *Data curation*: K.G., A. Katsuma, E.Y., T. Shofuda, K.F., K. Ichimura, Y. Matsushita, D. Kanematsu, A.S., J.H., T.I., Y. Kodama, M.M., N. Kijima, N. Kagawa, D. Keino, A. Mukasa, T. Suzuki, K. Yoshimoto, D. Kuga, K.H., S.Y., M. Kanamori, K. Yamasaki, K. Ishibashi, T.A., M.Y., R.I., A. Kawamura, S. Ohba, J.I., R.A., J.F., T.M., M.F., A. Muroi, K.S., A.H., Y.H., M. Nonaka, Y.‐S.P., Y. Kobayashi, T.H., Y. Miyairi, K. Yoshifuji, N.T., S. Oya, K.N., M. Nakada, Y. Nakano, M. Kambara, K.A., K.T., H.N., Y.S., R.S., T.W., and K.K. *Writing*: K.G., A. Katsuma, E.Y., Y.A., and Y. Kanemura. *Supervision*: Y. Kanemura, Y.A., S.S., H.A., H.S., I.D., M. Nagane, and R.N. *Project administration*: Y. Kanemura, Y.A., S.S., H.A., H.S., I.D., M. Nagane, and R.N. *Funding acquisition*: Y. Kanemura. All authors approved the final version of the manuscript.

## FUNDING INFORMATION

This research was supported by the Practical Research for Innovative Cancer Control from the Japan Agency for Medical Research and Development, AMED, and AMED under Grant Number JP19ck0106330.

## CONFLICT OF INTEREST STATEMENT

Yonehiro Kanemura is a recipient of a contract‐based research funding from K Pharma, Inc., and Kurabo Industries Ltd. for projects unrelated to this work. All other authors declare no competing interests related to this work.

## ETHICS STATEMENT

This nationwide multi‐institutional cohort was collected under multiple independent IRB approvals, and the consent frameworks did not permit the open release of detailed clinical information. However, de‐identified clinical data supporting the findings of this study are available from the corresponding author upon reasonable request, subject to approval by the Japan Pediatric Molecular Neuro‐Oncology Group and relevant institutional review boards.

## Supporting information


**Figure S1.** Integrated molecular diagnostics, profiling platforms, and methylation mapping in the JPMNG MB cohort.
**Figure S2.** Molecular subgroup and age distribution of medulloblastoma cases across multiple cohorts.
**Figure S3.** Overall survival (OS) analysis by age, histopathology, and metastatic status across all MB subgroups of the JPMNG cohort.
**Figure S4.** Copy number–based validation of MYB and E2F3 deletions using TaqMan Probe assays and methylation array data.
**Figure S5.** Kaplan–Meier survival analysis of the JPMNG cohort (WNT MB subgroup).
**Figure S6.** Kaplan–Meier survival analysis of the SHH MB subgroup in the JPMNG cohort.
**Figure S7.** Genomic alterations and survival analysis in Group 3 and Group 4 MBs.
**Figure S8.** Survival analysis and CNA‐based pattern stratification in Group 3 and Group 4 MBs.
**Figure S9.** Diagnostic algorithm for identifying WNT MB without methylation array analysis.

**Data S1.** Supporting Information.

**Table S1.** Detailed information on *TP53* mutation points and *TERT* promotor mutation in ClinVar.
**Table S2.** Genetic alteration frequencies in SHH‐MB: Northcott 2017 vs. JPMNG.
**Table S3.** Metastatic status and *MYC* amplification by age group in Group 3 MB.
**Table S4.** Distribution of radiotherapy modalities and CSI dose categories according to SEE‐6‐CNA status in Group 3 and Group 4 medulloblastoma.
**Table S5.** List of primary antibodies used.
**Table S6.** Primer sequences for PCR amplification of targeted regions in *CTNNB1*, *TERT*, and *TP53* genes.
**Table S7.** List of genes for copy number alteration analysis by SALSA MLPA.
**Table S8.** List of TaqMan copy number assays used for detection of monosomy 6 in WNT MB.

## Data Availability

The raw DNA methylation array data (IDAT files) generated in this study were deposited in the NCBI Gene Expression Omnibus (GEO) under the accession number GSE313522. In contrast, the clinical datasets and other individual‐level molecular data generated and analyzed in the current study are not publicly available due to ethical restrictions and concerns regarding patient privacy.
