# Correction to “Molecular and clinical features of a Japanese medulloblastoma cohort: Subgroup‐specific prognostic stratification using economical/accessible diagnostic methods”

**DOI:** 10.1111/bpa.70148

**Published:** 2026-09-21

Go K , Katsuma A , Yoshioka E , Shofuda T , Fukuoka K , Ichimura K , et al. Molecular and clinical features of a Japanese medulloblastoma cohort: Subgroup‐specific prognostic stratification using economical/accessible diagnostic methods. Brain Pathol 2026; 36(5):e70092. 10.1111/bpa.70092 PMC1342929641808532

The following corrections are required in the published article:

1. In the Figure 1B legend, “*p* = 0.0142” was incorrect. This should have read “*p* = 0.0149,” consistent with the main text and Figure 1B. Figure 1B itself is unchanged.

**FIGURE 2:**
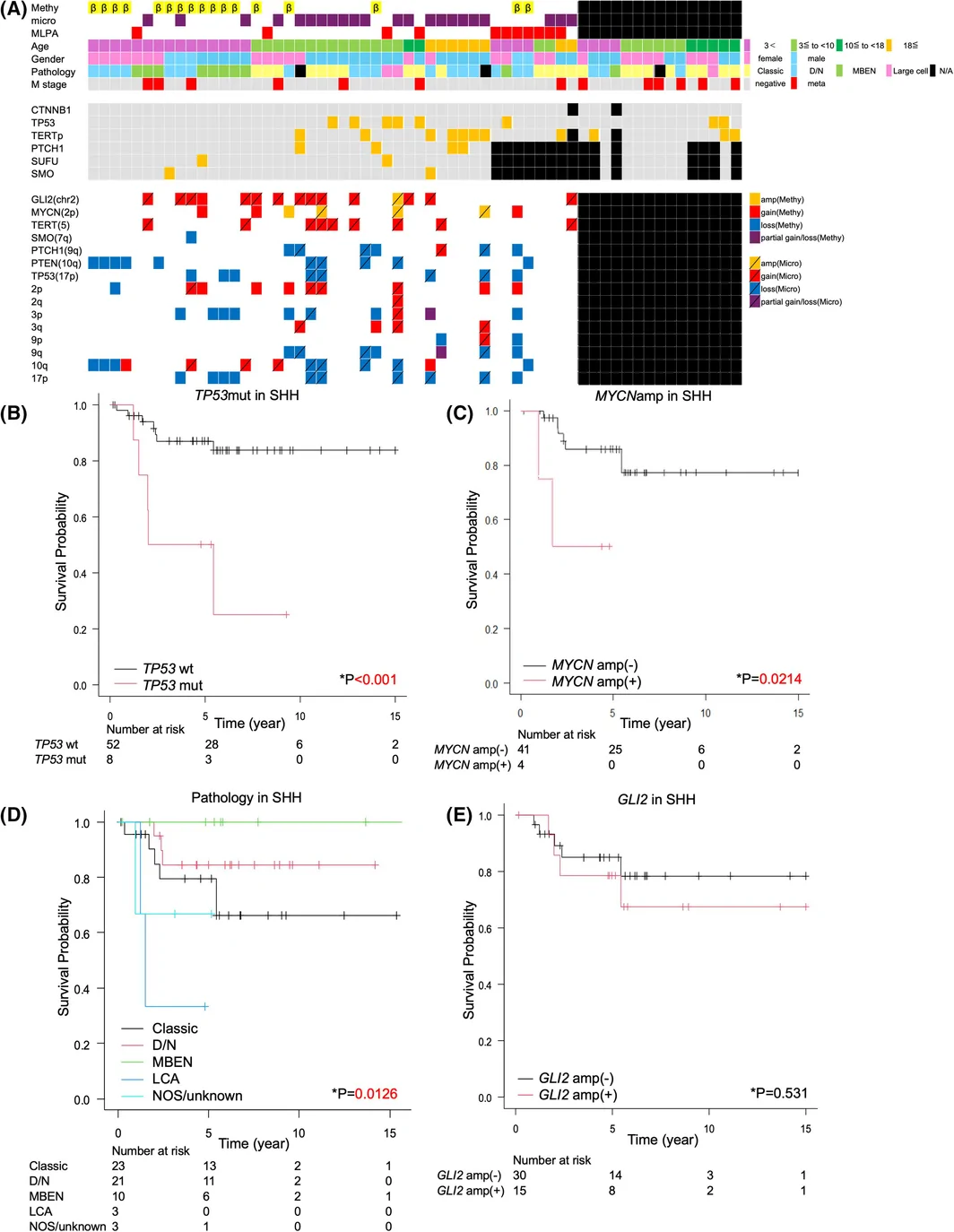

2 In Figure 2C, 15 cases for which *MYCN* amplification status was unavailable were inadvertently included in the non‐amplified group. The analysis has been repeated using only the 45 evaluable cases after excluding these 15 cases. The corrected analysis includes 41 *MYCN* non‐amplified cases and 4 *MYCN*‐amplified cases, with a log‐rank *p*‐value of 0.0214 (instead of 0.0374). Figure 2C should therefore be replaced with the corrected version. The association between *MYCN* amplification and poorer prognosis remains statistically significant, and the conclusion is unchanged.
3 On Page 6, in Section 3.3 of the Results section, the text “TP53 mutations, 12.1% (8/58)” was incorrect. This should have read “TP53 mutations, 13.3% (8/60).” TP53 status was evaluable in all 60 SHH cases, and the survival analysis in Figure 2B is unchanged.
4 On Page 8, in the first paragraph of Section 3.5 of the Results section, “classic CMB (96/101; 95.0%)” was incorrect. This should have read “classic CMB (95/101; 94.0%),” consistent with Table 1.
5 On Page 8, in the second paragraph of Section 3.5 of the Results section, the *p*‐value for the association between chromosome 11 loss and prognosis in Group 4 was incorrectly reported as “*p* = 0.0052.” This should have read “*p* = 0.045,” consistent with Figure 4C. The corrected *p*‐value remains statistically significant and the conclusion is unchanged.
6 On Page 14, in the 14th paragraph of the Discussion section, “Group 4 LCA cases (*n* = 5)” was incorrect. This should have read “Group 4 LCA cases (*n* = 3),” consistent with Table 1, the Results, and Figure 4D. The analysis and its interpretation are unchanged.

These corrections do not affect the principal conclusions or overall interpretation of the study.

We apologize for these errors.

