# Supplementary material for: Molecular and clinical features of a Japanese medulloblastoma cohort: Subgroup‐specific prognostic stratification using economical/accessible diagnostic methods
Source: Brain Pathol. 2026 Mar 11;36(5):e70092. doi: 10.1111/bpa.70092 (PMC13429296; doi:10.1111/bpa.70092)
Supplement: Supplementary file 1 — Figure S1. Integrated molecular diagnostics, profiling platforms, and methylation mapping in the JPMNG MB cohort. Figure S2. Molecular subgroup and age distribution of medulloblastoma cases across multiple cohorts. Figure S3. Overall survival (OS) analysis by age, histopathology, and metastatic status across all MB subgroups of the JPMNG cohort. Figure S4. Copy number–based validation of MYB and E2F3 deletions using TaqMan Probe assays and methylation array data. Figure S5. Kaplan–Meier survival analysis of the JPMNG cohort (WNT MB subgroup). Figure S6. Kaplan–Meier survival analysis of the SHH MB subgroup in the JPMNG cohort. Figure S7. Genomic alterations and survival analysis in Group 3 and Group 4 MBs. Figure S8. Survival analysis and CNA‐based pattern stratification in Group 3 and Group 4 MBs. Figure S9. Diagnostic algorithm for identifying WNT MB without methylation array analysis. [file BPA-36-e70092-s003.docx]

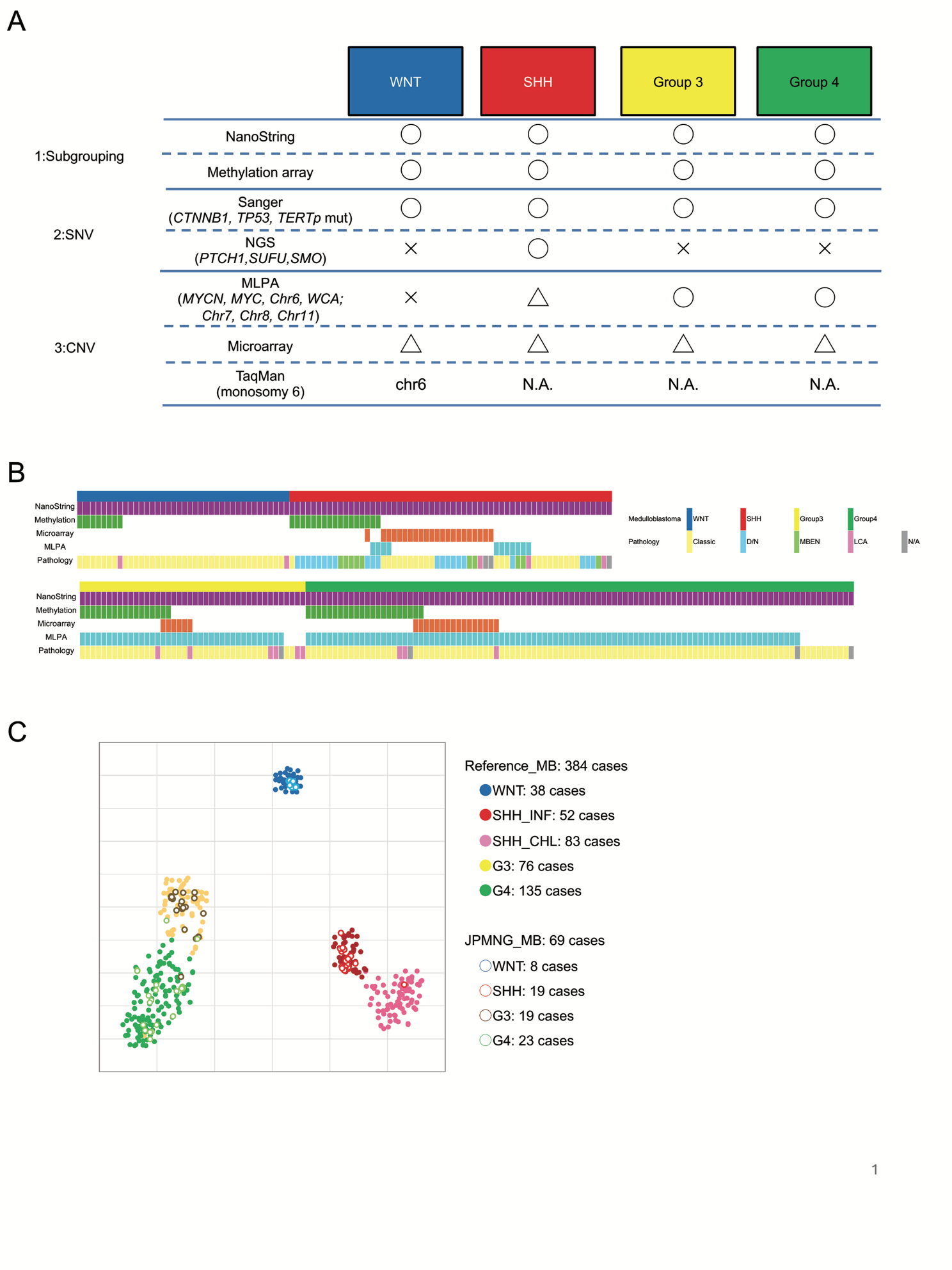


**Supplementary Figure 1. Integrated Molecular Diagnostics, Profiling Platforms, and Methylation Mapping in the JPMNG MB Cohort**

(A) Summary of the molecular diagnostic platforms used for subgroup classification in the JPMNG cohort (n = 242).

Four molecular subgroups—WNT, SHH, Group 3, and Group 4—were identified using a multi-platform approach.

(1) Subgroup assignment via NanoString gene expression profiling and methylation array (DKFZ classifier).

(2) Detection of single nucleotide variants (SNVs) using Sanger sequencing and next-generation sequencing (NGS);

(3) CNAs analysis using MLPA, microarray, and quantitative PCR (TaqMan).

Symbol annotations indicate the applicability and informativeness of each assay for the corresponding subgroup: ○ = performed/informative; △= partially informative; × = not applicable. Monosomy 6 analysis (chr6) was specific to the WNT subgroup.

(B) Overview of molecular profiling platforms and clinicopathological characteristics across MB subgroups.

Each column represents an individual MB case stratified by the molecular subgroup, as indicated in the top row. The rows denote the status and availability of molecular profiling methods—NanoString, DNA methylation array, CNV microarray, and MLPA—as well as key clinicopathological features, including histological subtype and relevant annotations.

(C) t-distributed stochastic neighbor embedding (t-SNE) plot of MB methylation data from the JPMNG cohort alongside reference data for MBs. 11 Cases from the JPMNG cohort are represented by open-circle symbols to distinguish them from the reference set, which is depicted using filled circle symbols. Samples with a calibrated score below 0.9 from the DKFZ methylation classifier were annotated with their case number, assigned subtype (numerical), and corresponding calibrated score.





**Supplementary Figure 2. Molecular Subgroup and Age Distribution of Medulloblastoma Cases Across Multiple Cohorts**

(A) Distribution of MB molecular subgroups in the JPMNG cohort (n = 242) compared with representative international cohorts from Korea, India, MAGIC, and China. 3,20-22

(B) Age distribution of patients in the JPMNG cohort.

(C) Age-stratified distribution of molecular subgroups in the JPMNG cohort.

(D) Subgroup distribution among infants (<3 years) across the selected cohorts.

(E) Subgroup distribution among pediatric cases (ages 3 ≤ to <10 years) across selected cohorts.

(F) Subgroup distribution among adult cases (≥18 years) across the selected cohorts.





**Supplementary Figure 3. Overall Survival (OS) Analysis by Age, Histopathology, and Metastatic Status Across All MB Subgroups of the JPMNG cohort**

(A) Kaplan–Meier OS curves for infants (age <3 years) across all molecular subgroups.

(B) OS curves for pediatric cases (ages 3 to <10 years) in all subgroups.

(C) OS curves for adolescent cases (aged 10 to <18 years) across all subgroups.

(D) OS curves for adult patients (age ≥18 years) across all subgroups.

(E) Combined OS curves for all age groups within the cohort.

(F) Kaplan–Meier analysis of OS stratified by histopathological classification across all subgroups.

(G) OS curves based on metastatic status in all subgroups.


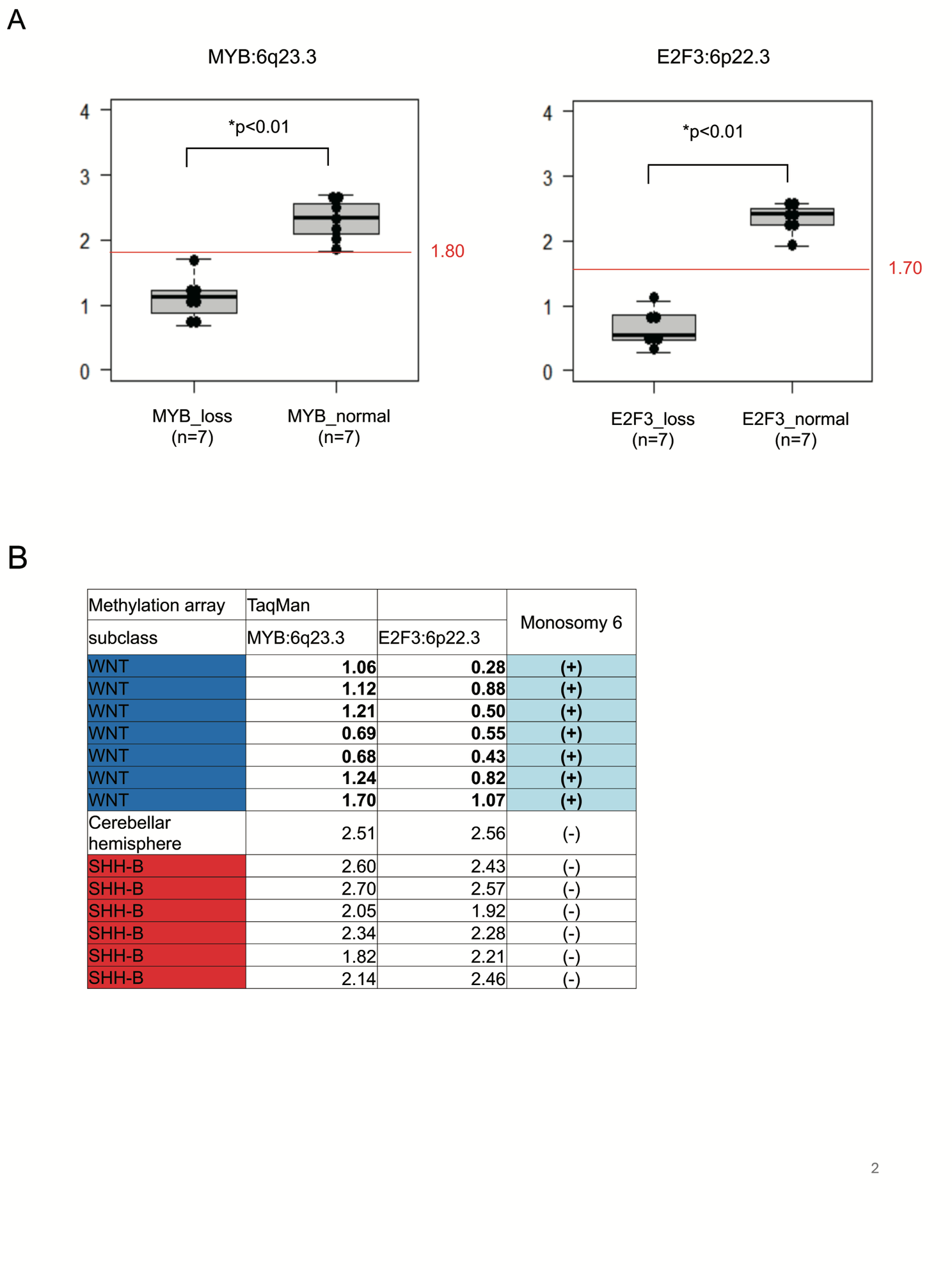


**Supplementary Figure 4. Copy Number–Based Validation of MYB and E2F3 Deletions Using TaqMan Probe Assays and Methylation Array Data**

(A) TaqMan probe assays were used to quantify the copy number values of MYB (6q23.3) and E2F3 (6p22.3) in tumor samples. Based on methylation-derived copy number profiles, tumors were categorized into “loss” (n = 7) or “normal” (n = 7) groups. Empirical thresholds were set at 1.80 for MYB and 1.70 for E2F3 (indicated by red lines). Samples in the “loss” category showed significantly reduced copy number values compared to the “normal” group (p < 0.01 for both genes). These thresholds were applied for classification throughout the study period.

(B) Validation of copy number findings in additional subgroup-defined samples. All WNT MB cases demonstrated concurrent deletion of MYB and E2F3, accompanied by monosomy 6. In contrast, SHH subgroup cases and those originating from the cerebellar hemisphere exhibited normal or elevated copy numbers, supporting the presence of subgroup-specific CNAs profiles.


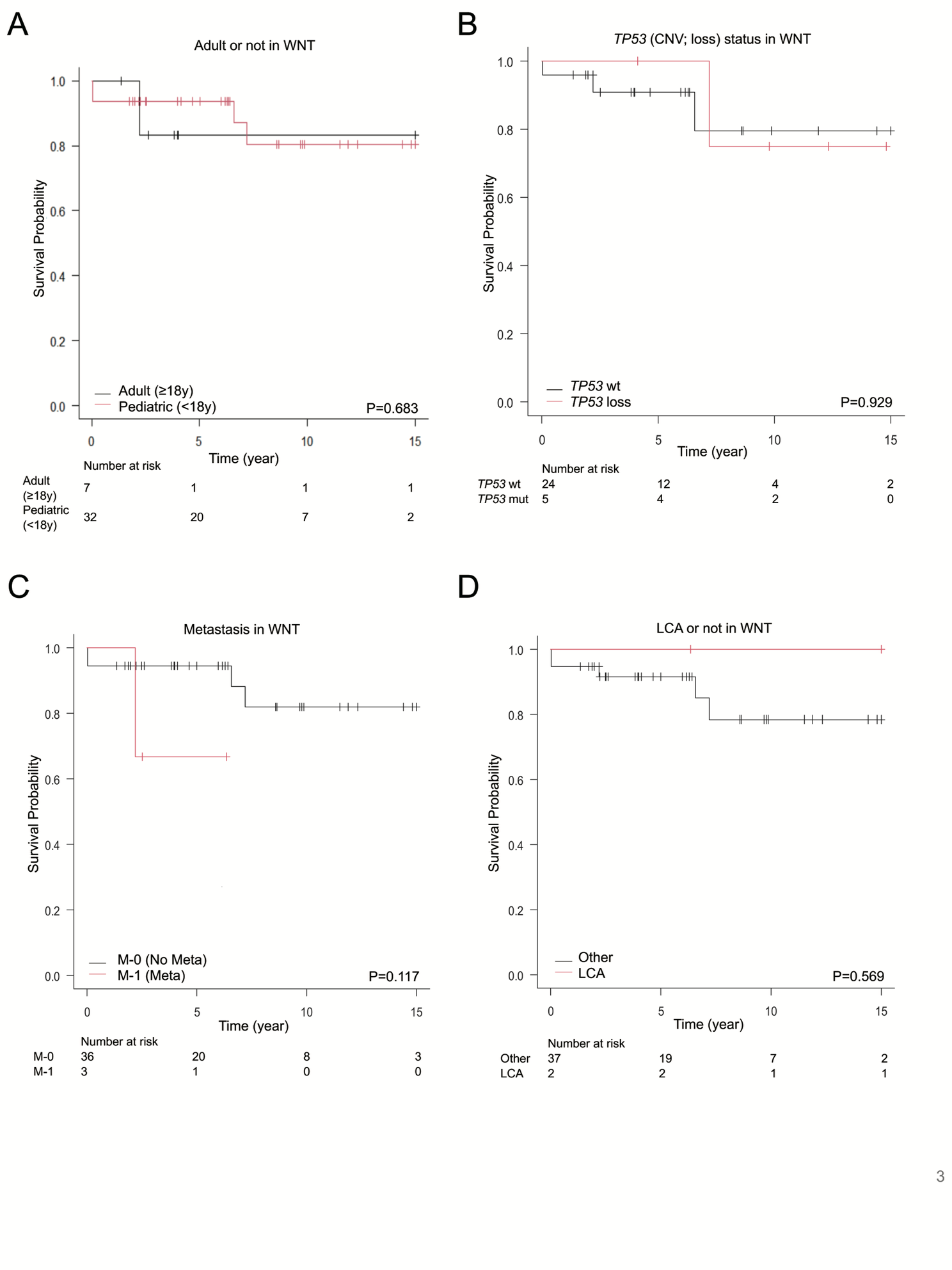


**Supplementary Figure 5. Kaplan–Meier Survival Analysis of the JPMNG Cohort (WNT MB Subgroup)**

(A) OS curves comparing adult and non-adult patients within the WNT MB subgroup.

(B) Survival analysis stratified by TP53 mutation status in WNT-MB.

(C) Kaplan–Meier curves illustrating survival based on the metastatic status of WNT MB.

(D) OS curves according to the presence or absence of large cell/anaplastic (LCA) histology in WNT MB.





**Supplementary Figure 6. Kaplan–Meier Survival Analysis of the SHH MB Subgroup in the JPMNG Cohort**

(A) OS curves stratified by age group within the SHH MB subgroups.

(B) Survival analysis based on TERT promoter mutation status in SHH MB.

(C) Kaplan–Meier curves depicting OS according to TP53 (17p) loss in SHH MB.

(D) OS curves for PTCH1 mutation status in SHH MB.

(E) Survival analysis based on SMO mutation status in SHH MB.

(F) Kaplan–Meier curves showing OS stratified by SUFU mutation status in SHH MB.


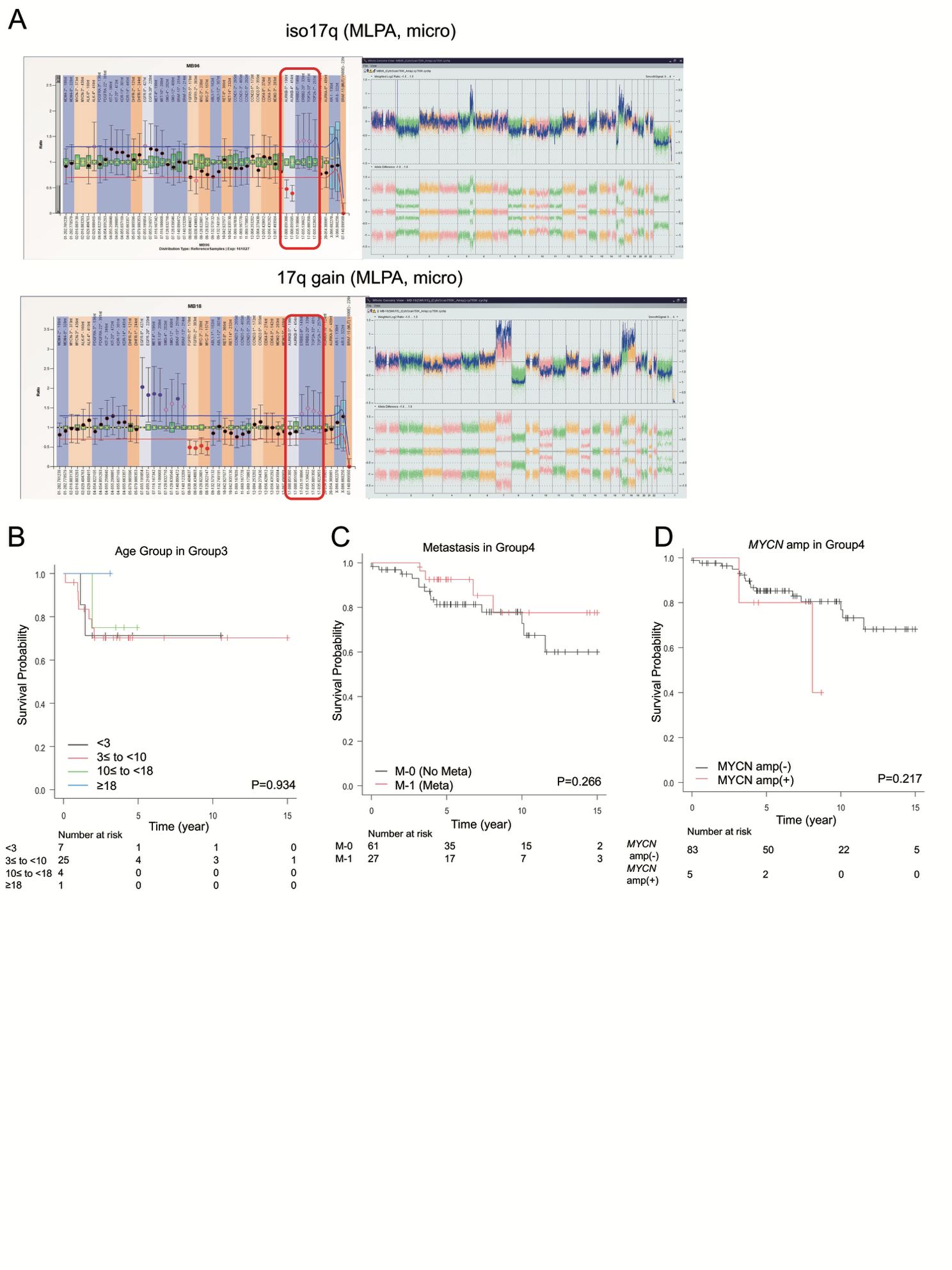


**Supplementary Figure 7. Genomic Alterations and Survival Analysis in Group 3 and Group 4 MBs**

(A) Copy number profiles assessed using MLPA (left panels) and CNV microarray (right panels) from matched tumor samples.

Upper panel: Concurrent loss of 17p and gain of 17q (highlighted in red) indicate the presence of isochromosome 17q. This alteration was consistently detected by both MLPA and CNV microarrays.

Lower panel: Isolated copy number gain of 17q with preserved 17p copy number, confirming non-isochromosome 17q amplification using both platforms.

(B) Kaplan–Meier OS curves stratified by age group for Group 3 MBs.

(C) Survival analysis based on metastatic status in Group 4 MBs.

(D) OS curves according to the MYCN amplification status in Group 4 MBs.


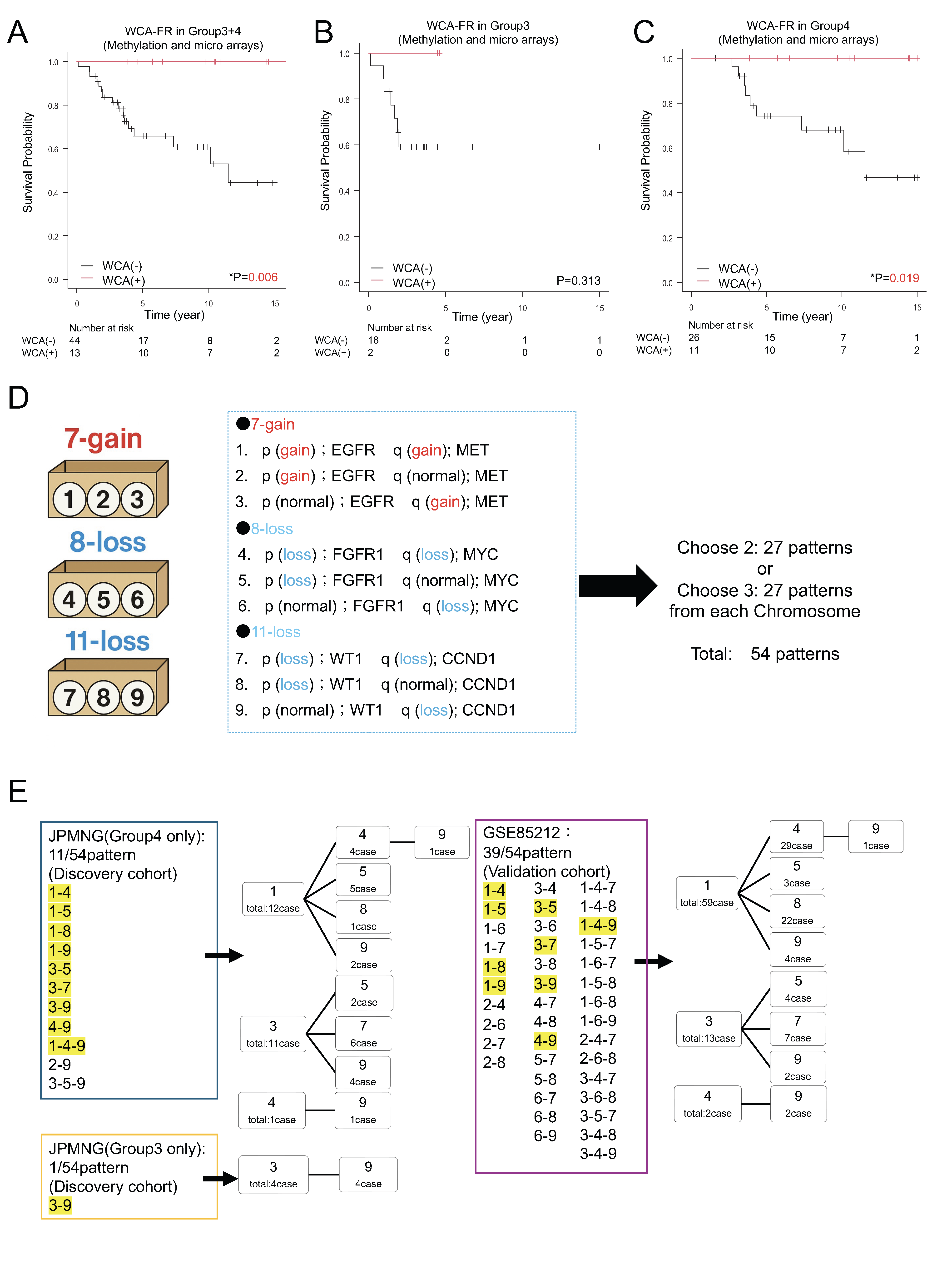


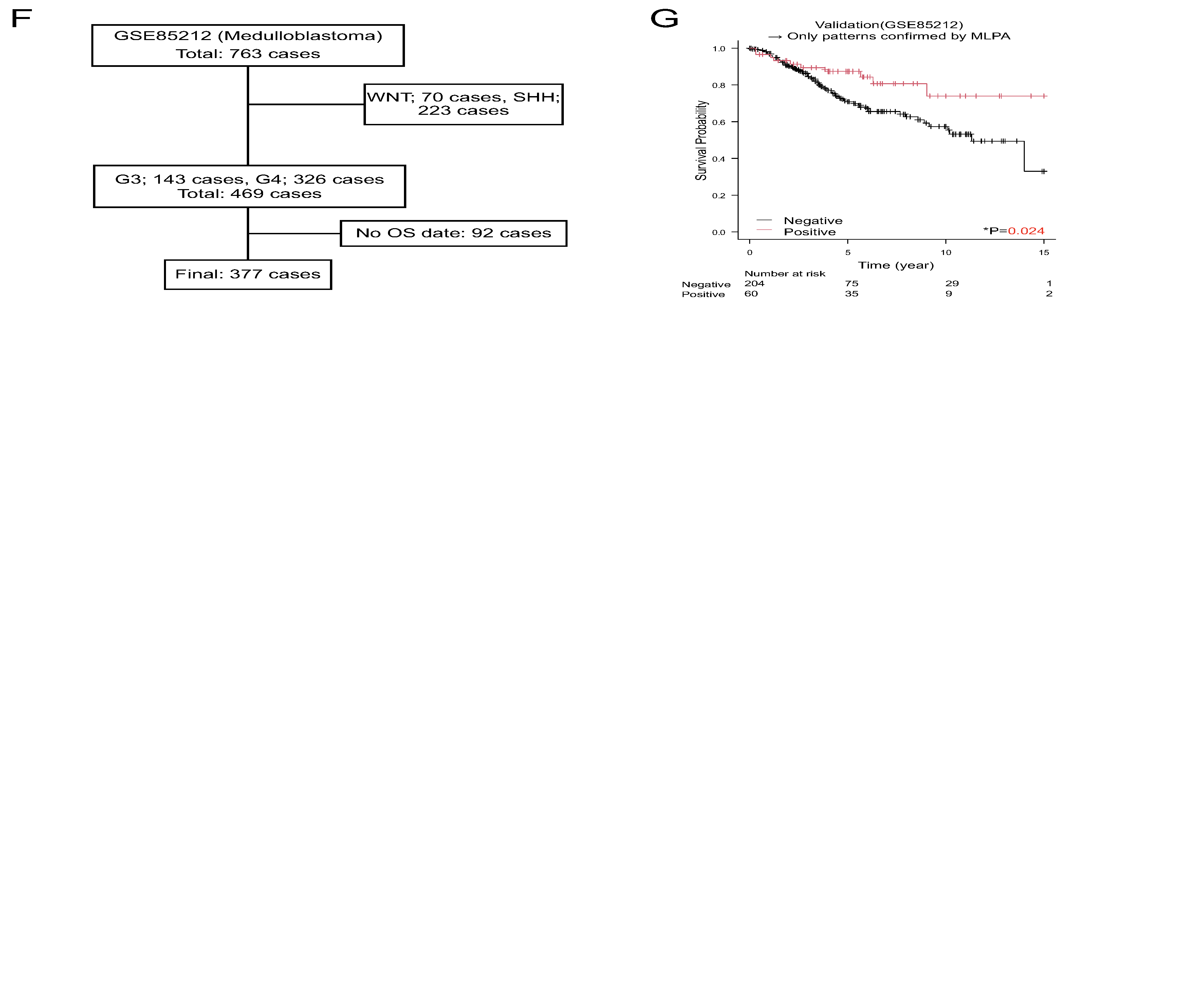


**Supplementary Figure 8: Survival Analysis and CNA-Based Pattern Stratification in Group 3 and Group 4 MBs**

(A) Kaplan–Meier survival analysis of WCA-FR–positive [ (WCA +)] and WCA-FR–negative [ (WCA–)] cases in Groups 3 and 4. Only cases assessed using DNA methylation arrays or CNV microarrays were included.

(B) OS curves based on WCA-FR status within Group 3 MBs.

(C) OS curves based on WCA-FR status within Group 4 of the MBs.

(D) Conceptual overview of the SEE-6-CNA classification framework. Copy number profiles were assessed across six loci located on chromosomes 7 (EGFR, MET), 8 (FGFR1, MYC), and 11 (WT1, CCND1), each of which was evaluated for gain, loss, or neutral status. By combining these patterns across two or three chromosomes, 54 unique CNA combinations were defined and used to stratify non-WNT/non-SHH MBs.

(E) Distribution and reproducibility of probe patterns in Groups 3 and 4. Of the 54 predefined CNA patterns, 12 were detected in the JPMNG discovery cohort (left panel) and 39 in the GSE85212 validation cohort (middle panel). Hierarchical tree diagrams illustrate sample clustering based on shared pattern combinations. The yellow nodes highlight the probe patterns observed in both cohorts, demonstrating reproducibility. Notably, pattern 3-9 was exclusively identified in Group 3 within the JPMNG cohort, highlighting its subgroup-specific association.

(F) Summary of cohort characteristics of the GSE85212 methylation dataset. The dataset included 763 medulloblastoma cases, of which 469 (61.5%) had complete molecular subgroup data for Groups 3 and 4. After excluding 92 cases lacking overall survival (OS) information, 377 cases (49.4%) with both molecular classification and clinical outcome data were included in the final analysis.

(G) Kaplan–Meier survival analysis based on the presence or absence of characteristic probe patterns identified in the JPMNG cohort. Nine specific CNA patterns were used to classify the cases as pattern-positive. Survival comparisons between the pattern-positive and pattern-negative groups were performed to evaluate prognostic significance.





**Supplementary Figure 9. Diagnostic Algorithm for Identifying WNT MB Without Methylation Array Analysis**

A preliminary classification of WNT MB was made using NanoString gene expression assay results. A definitive diagnosis is confirmed when at least one of the following supporting features is present: nuclear β-catenin positivity on immunohistochemistry, CTNNB1 mutation, or monosomy 6. Cases lacking these additional markers are excluded from the WNT MB classification and are recommended for further molecular testing to refine subgroup assignment.
