# Supplementary material for: Molecular and clinical features of a Japanese medulloblastoma cohort: Subgroup‐specific prognostic stratification using economical/accessible diagnostic methods
Source: Brain Pathol. 2026 Mar 11;36(5):e70092. doi: 10.1111/bpa.70092 (PMC13429296; doi:10.1111/bpa.70092)
Supplement: Supplementary file 2 — Data S1. Supporting Information. [file BPA-36-e70092-s002.docx]

**Supplementary information**

Immunohistochemistry

Surgical specimens were fixed in buffered formalin and embedded in paraffin to generate formalin-fixed, paraffin-embedded (FFPE) samples. Tissue sections were prepared and subjected to hematoxylin and eosin (H&E) staining or immunohistochemical analysis using primary antibodies listed in Supplementary Table 5. Immunostaining was performed on the Ventana BenchMark ULTRA platform (Roche Diagnostics, Basel, Switzerland), with endogenous peroxidase activity blocked. Signal detection was performed using the UltraView DAB detection kit, and nuclear counterstaining was performed with hematoxylin on the same system. Immunohistochemical data from several cases were obtained using specimens prepared at each participating institution using comparable methodologies.

DNA/RNA extraction

Total RNA was extracted using QIAzol Lysis Reagent (Qiagen, Valencia, CA, USA) according to the manufacturer's instructions. Genomic DNA was isolated using the NucleoSpin Tissue Kit (Macherey-Nagel, Düren, Germany). For tissue samples, total RNA and genomic DNA were purified using the Presto DNA/RNA Extraction Kit (Geneaid Biotech Ltd., New Taipei City, Taiwan), whereas FFPE samples were processed using the FormaPure XL Total Kit (Beckman Coulter, Inc., Brea, CA, USA), following the respective manufacturer’s protocols.

Gene expression analysis by nCounter System

Target RNA (100 ng) was hybridized using NanoString technology or nCounter Elements Technology at 65°C for 16 h, following the manufacturer’s protocol (NanoString Technologies, Inc., Seattle, WA, USA). Hybridization reactions were performed using the nCounter Prep Station (NanoString Technologies, Inc.), and expression profiling of 22 medulloblastoma subgroup-specific genes and three housekeeping genes was performed on the nCounter Digital Analyzer (NanoString Technologies, Inc.) by quantifying reporter signal counts. The extracted data were subsequently used for medulloblastoma subgroup classification using R software ^[1]^.

Genome-wide DNA methylation analysis

Comprehensive methylation analysis was conducted using the Illumina Infinium HumanMethylationEPIC (EPIC array) or HumanMethylation450k (450k array) BeadChip array (Illumina, San Diego, CA, USA), which includes 866,238 or 485,512 CpG sites for analysis, respectively, as previously described. ^[2]^ Raw methylation data (idat files) were uploaded to the German Cancer Center's website [https://www.molecularneuropathology.org/mnp] for classification using the DKFZ classifier (ver. 12.5).  ^[3]^

Sanger sequencing

Regions of interest for each gene were amplified from genomic DNA using gene-specific primers (Supplementary Table 6) with AmpliTaq Gold 360 Master Mix (Thermo Fisher Scientific) on either a GeneAmp PCR System 9700 or a Veriti Thermal Cycler (both from Thermo Fisher Scientific). PCR products were purified using ExoSAP-IT (Thermo Fisher Scientific) and subsequently sequenced with the corresponding sequencing primers (Supplementary Table 6) using the BigDye® Terminator v1.1 Cycle Sequencing Kit (Thermo Fisher Scientific). Sequencing was performed using an ABI 3130xL or 3500 Genetic Analyzer (Thermo Fisher Scientific).

Next-generation sequencing analysis

Primers for target sequencing were designed by Thermo Fisher Scientific based on the specified genes of interest. DNA libraries were constructed using the Ion AmpliSeq Library Kit 2.0 (Thermo Fisher Scientific) following the manufacturer's instructions. Library quantification was performed using the Agilent High-Sensitivity DNA Kit on an Agilent 2100 Bioanalyzer (Agilent Technologies, Santa Clara, CA, USA). Sequencing was performed using the Ion Proton™ system (Thermo Fisher Scientific). Amplicon sequences were aligned to the human reference genome (hg19) using Torrent Variant Caller (Thermo Fisher Scientific).

CNV Microarray analysis

Genomic DNA was hybridized to either the Affymetrix CytoScan® 750K Array or CytoScan HD Array (Thermo Fisher Scientific) in accordance with the manufacturer’s protocol. Following a 16-hour incubation at 50°C, the arrays were washed and stained using a Fluidics Station (Thermo Fisher Scientific) and scanned with a GeneChip Scanner (Thermo Fisher Scientific). Array CEL files were analyzed using the Chromosome Analysis Suite software (Thermo Fisher Scientific) with a high-resolution filter setting to detect copy number variations.

MLPA (Multiplex Ligation-dependent Probe Amplification)

MLPA analysis was performed using SALSA MLPA Kits P175 (version A3) and P294 (version C1), following the manufacturer's instructions (MRC Holland, Amsterdam, Netherlands). The P175 kit targets genes suspected of copy number gain or amplification in tumor cells, whereas the P294 kit targets genes suspected of copy number loss (Supplementary Table 7).

Amplification products were separated on an ABI 3130xL Genetic Analyzer (Applied Biosystems, Foster City, CA, USA) and quantified using the GeneMapper 5.0 software (Applied Biosystems). Quantitative data for each target region were analyzed using Coffalyser software, version 140721.1958 (MRC Holland; [www.coffalyser.net](https://www.coffalyser.net/)), in which the relative probe signals were compared with those of normal control samples.

The DNA copy number of each gene was determined by averaging the values of all corresponding probes. Genes were classified based on the following thresholds:

- **Amplification (Amp):** ≥ 2.0
- **Gain:** > 1.3 and ≤ 2.0
- **Neutral:** > 0.5 and ≤ 1.3
- **Loss:** ≤ 0.5

Data from poor-quality DNA samples, inconsistent probe values, and aberrant reference gene signals were excluded from the survival analysis.

Copy number analysis by TaqMan Copy Number Assays

Copy number analysis of chromosome 6 was performed using TaqMan Copy Number Assays (Thermo Fisher Scientific) on a QuantStudio 12K Flex Real-Time PCR System (Thermo Fisher Scientific). The details of each assay are provided in Supplementary Table 8.

Human genomic DNA controls—Female (G1521), Male (G1471), and Mixed (G304A)—were obtained from Promega (Madison, WI, USA). Ten nanograms of genomic DNA from each sample and control were subjected to real-time PCR using both target-specific TaqMan Copy Number Assays and a reference assay. Copy number values were calculated based on ΔCt normalization relative to the reference assay and compared with control DNA samples to determine relative copy number estimates.

The copy number was calculated using the following formula:
**Copy number** = 2 × 2^−[(Ct_Target(Sample) − Ct_Reference(Sample)) − (Ct_Target(Control) − Ct_Reference(Control))]

References

1.　R Core Team. R: A Language and Environment for Statistical Computing [Computer Program]. Version 4.30. Vienna, Austria: R Foundation for Statistical Computing; 2023.

2.　Fukuoka K, Kurihara J, Shofuda T, et al. Subtyping of Group 3/4 medulloblastoma as a potential prognostic biomarker among patients treated with reduced dose of craniospinal irradiation: a Japanese Pediatric Molecular Neuro-Oncology Group study. Acta Neuropathol Commun. 2023; 11(1):153.

3.　Casanova G, David K, et al. Diagnostic impact of DNA methylation classification in adult and pediatric central nervous system tumors. Sci Rep. 2025;15:87079. doi:10.1038/s41598-025-87079-4.
