# Supplementary material for: Molecular and clinical features of a Japanese medulloblastoma cohort: Subgroup‐specific prognostic stratification using economical/accessible diagnostic methods
Source: Brain Pathol. 2026 Mar 11;36(5):e70092. doi: 10.1111/bpa.70092 (PMC13429296; doi:10.1111/bpa.70092)
Supplement: Supplementary file 3 — Table S1. Detailed information on TP53 mutation points and TERT promotor mutation in ClinVar. Table S2. Genetic alteration frequencies in SHH‐MB: Northcott 2017 vs. JPMNG. Table S3. Metastatic status and MYC amplification by age group in Group 3 MB. Table S4. Distribution of radiotherapy modalities and CSI dose categories according to SEE‐6‐CNA status in Group 3 and Group 4 medulloblastoma. Table S5. List of primary antibodies used. Table S6. Primer sequences for PCR amplification of targeted regions in CTNNB1, TERT, and TP53 genes. Table S7. List of genes for copy number alteration analysis by SALSA MLPA. Table S8. List of TaqMan copy number assays used for detection of monosomy 6 in WNT MB. [file BPA-36-e70092-s001.docx]

Supplementary Table 1

Detailed information on *TP53* mutation points and *TERT* promotor mutation in ClinVar

| *TP53* | Pathogenic | Likely pathogenic | *TERT* | Cases |
| --- | --- | --- | --- | --- |
|  | c.743G>A, p.R248Q | c.535delC: p.H179Mfs*68 | C228T c.-124C>T [homo] | 8 |
|  |  |  | *c.-124C>C/T [hetero] | 3 |
|  | c.659A>G, p.Y220C |  |  |  |
|  |  |  |  |  |
|  | c.817C>T, p.R273C  (Substitution-missense) |  |  |  |
|  | c.708delC, p.Y236*  c.824G>T, p.C275F |  |  |  |
|  | c.742C>T, p.R248W |  |  |  |
|  | c.376-1G>A, p.? |  |  |  |
|  | c.733G>A, p.G245S |  |  |  |

*TP53* and *TERT* promoter mutations were detected in the SHH-subgroup medulloblastoma cases in the JPMNG cohort. A total of 8 cases harbored *TP53* mutations, with 9 distinct variants identified. All *TP53* variants were classified as pathogenic or likely pathogenic based on ClinVar annotations. The notation “*c.-124C>C/T” in the *TERT* promoter row indicates heterozygous variants in which both C>C and C>T alleles were detected.

Supplementary Table 2

Genetic Alteration Frequencies in SHH-MB: Northcott 2017 vs. JPMNG

|  |  | Northcott. 2017 | JPMNG |
| --- | --- | --- | --- |
| SHH |  | 30.0% | 24.8% |
| SNV | *PITCH* | 43.0% | 20.0% (9/45) |
|  | *TERTp* | 39.0% | 19.0% (11/58) |
|  | *TP53* | 13.0% | 12.1% (8/58) |
|  | *SUFU* | 10.0% | 4.4% (2/45) |
|  | *SMO* | 9.0% | 4.4% (2/45) |
| CNA | *MYCN* amp | 7.0% | 8.9% (4/45) |
|  | *PTEN* loss | 7.0% | 22.2% (10/45) |

Compares the frequency of somatic pathogenic SNVs and CNAs in JPMNG SHH MB between the present JPMNG study and the series reported by Northcott et al. (Nature, 2017). Comparable trends were observed for *TP53* and *MYCN* amplifications; however, the frequencies were generally low. A comparison of the two cohorts revealed that the proportion of *PTEN* loss was higher in the JPMNG cohort.

Supplementary Table 3

Metastatic status and *MYC* amplification by age group in Group 3 MB

| Age group | Total cases (n) | Metastatic disease n (%) | *MYC* amplification n (%) |
| --- | --- | --- | --- |
| **< 3 years** | 7 | 5 (71.4) | 1 (14.3) |
| **3–10 years** | 25 | 13 (52.0) | 5 (20.0) |
| **10–18 years** | 4 | 1 (25.0) | 0 (0.0) |
| **> 18 years** | 1 | 0 (0.0) | 0 (0.0) |

This is a summary of the analysis results for the 37 cases with available MLPA‑based copy‑number data. Metastatic disease indicates metastasis at diagnosis. All metastatic infants (<3 years) received CSI; among these (n=5), one was a survivor.

Supplementary Table 4.

 Distribution of radiotherapy modalities and CSI dose categories according to SEE-6-CNA status in Group 3 and Group 4 medulloblastoma.

| Subgroup / SEE-6-CNA status | n (total) | No RT  (%) | Focal RT only  (%) | CSI  (%) | Low-dose CSI (%) | Standard-dose CSI (%) | High-dose CSI (%) | Unknown (%) |
| --- | --- | --- | --- | --- | --- | --- | --- | --- |
| Group 3, positive | 4 | 1 (25.0) | 0 (0.0) | 3 (75.0) | 0 (0.0) | 3 (75.0) | 0 (0.0) | 0 (0.0) |
| Group 3, negative | 33 | 3 (9.1) | 0 (0.0) | 29 (87.9) | 6 (18.2) | 19 (57.6) | 4 (12.1) | 1 (3.0) |
| Group 4, positive | 24 | 0 (0.0) | 0 (0.0) | 23 (95.8) | 3 (12.5) | 15 (62.5) | 5 (20.8) | 1 (4.2) |
| Group 4, negative | 65 | 3 (4.6) | 3 (4.6) | 59 (90.8) | 11 (16.9) | 34 (52.3) | 17 (26.2) | 0 (0.0) |

Low-dose CSI：< 23.4Gy, Standard-dose CSI：23.4Gy～< 36Gy, High-dose CSI：≥ 36Gy

Summary of radiotherapy patterns in non-WNT/non-SHH medulloblastoma, stratified by molecular subgroup (Group 3 vs Group 4) and SEE-6-CNA status (positive vs negative). For each subgroup, the table shows the number and proportion of patients who received no radiotherapy, focal radiotherapy only, or craniospinal irradiation (CSI), as well as the distribution of the CSI dose categories. CSI doses were classified as low-dose (<23.4 Gy), standard-dose (23.4–<36 Gy), and high-dose (≥36 Gy). The percentages were calculated relative to the total number of patients in each row.

Supplementary Table 5

List of primary antibodies used

| Antigen | Clone | Supplier |
| --- | --- | --- |
| ki-67 | 30-9 | Roche |
| synaptophysin | MRQ-40 | CELL MARQUE |
| GFAP | EP672Y | CELL MARQUE |
| INI-1 | 25/BAF47 | BD |
| EMA | E29 | Roche |
| NF | RMdO-20 | BioLegend |
| β-catenin | 14 | CELL MARQUE |
| p53 | DO-7 | Roche |

Supplementary Table 6

Primer Sequences for PCR Amplification of Targeted Regions in *CTNNB1, TERT,* and *TP53* Genes

| Target Region | Forward primer (5’ to 3’) | Reverse primer (5’ to 3’) |
| --- | --- | --- |
| *CTNNB1* exon 3 | TGGAACCAGACAGAAAAGCG | ACAGGACTTGGGAGGTATCC |
| *TERT* promoter | CCTGCCCCTTCACCTTCCAG | AGGACGCAGCGCTGCCTGAA |
| *TP53* exon 2–4 | CAGGAGTGCTTGGGTTGTGG | AGAAATGCAGGGGGATACGGC |
| *TP53* exon 5–6 | TGCCCTGACTTTCAACTCTG | CTACTGCTCACCCGGAGG |
| *TP53* exon 7–9 | CCACAGGTCTCCCCAAGG | GTTAGCTACAACCAGGAGCCA |
| *TP53* exon 10–11 | ATGCATGTTGCTTTTGTACCG | TGTTCTGACGCACACCTAT |

Supplementary Table 7

List of genes for copy number alteration analysis by SALSA MLPA

|  | Gene | Chr.band |  | Gene | Chr.band |
| --- | --- | --- | --- | --- | --- |
| P175 | *MDM4* | 01q32.1 | P294 | *TNFRSF4* | 01p36.33 |
|  | *MYCN* | 02p24.3 |  | *PRDM16* | 01p36.32 |
|  | *ALK* | 02p23.2 |  | *CHD5* | 01p36.31 |
|  | *PDGFRA* | 04q12 |  | *CAMTA1* | 01p36.23 |
|  | *KIT* | 04q12 |  | *KIF1B-2* | 01p36.22 |
|  | *KDR* | 04q12 |  | *VHL* | 03p25.3 |
|  | *DHFR* | 05q14.1 |  | *FHIT* | 03p14.2 |
|  | *EGFR* | 07p11.2 |  | *APC* | 05q22.2 |
|  | *MET* | 07q31.2 |  | *CDKN2A* | 09p21.3 |
|  | *SMO* | 07q32.1 |  | *CDKN2B* | 09p21.3 |
|  | *BRAF* | 07q34 |  | *PTCH1* | 09q22.32 |
|  | *FGFR1* | 08p12 |  | *TSC1* | 09q34.13 |
|  | *MYC* | 08q24.21 |  | *PTEN* | 10q23.31 |
|  | *ABL1* | 09q34.12 |  | *WT1* | 11p13 |
|  | *RET* | 10q11.21 |  | *BRCA2* | 13q13.1 |
|  | *CCND1* | 11q13.2 |  | *RB1* | 13q14.2 |
|  | *CCND2* | 12p13.32 |  | *MIR15A* | 13q14.3 |
|  | *CDK4* | 12q14.1 |  | *DLEU1* | 13q14.3 |
|  | *MDM2* | 12q15 |  | *TSC2* | 16p13.3 |
|  | *AURKB* | 17p13.1 |  | *TP53* | 17p13.1 |
|  | *ERBB2* | 17q12 |  | *NF1* | 17q11.2 |
|  | *TOP2A* | 17q21.2 |  | *BRCA1* | 17q21.31 |
|  | *AURKA* | 20q13.2 |  | *SMAD4* | 18q21.2 |
|  | *AR-1* | Xq12 |  | *STK11* | 19p13.3 |
|  |  |  |  | *FKBP8* | 19p13.11 |
|  |  |  |  | *SMARCB1* | 22q11.23 |
|  |  |  |  | *AMER1* | Xq11.1 |

Supplementary Table 8

List of TaqMan Copy Number Assays Used for Detection of Monosomy 6 in WNT MB

| Gene Symbol | Assay ID | Cytoband | Reporter | Quencher |
| --- | --- | --- | --- | --- |
| *MYB* | Hs00840139_cn | 6q23.3 | FAM dye | NFQ-MGB |
| *VEGFA* | Hs02336545_cn | 6p21.1 | FAM dye | NFQ-MGB |
| *E2F3* | Hs01100244_cn | 6p22.3 | FAM dye | NFQ-MGB |
| TaqMan Copy Number Reference Assays: RNase P | | | VIC dye | NFQ-MGB |
